# On State-Space Reduction in Multi-Strain Pathogen Models, with an Application to Antigenic Drift in Influenza A

**DOI:** 10.1371/journal.pcbi.0030159

**Published:** 2007-08-17

**Authors:** Sergey Kryazhimskiy, Ulf Dieckmann, Simon A Levin, Jonathan Dushoff

**Affiliations:** 1 Program in Applied and Computational Mathematics, Princeton University, Princeton, New Jersey, United States of America; 2 Evolution and Ecology Program, International Institute for Applied Systems Analysis, Laxenburg, Austria; 3 Department of Ecology and Evolutionary Biology, Princeton University, Princeton, New Jersey, United States of America; 4 Fogarty International Center, National Institute of Health, Bethesda, Maryland, United States of America; Johns Hopkins University, United States of America

## Abstract

Many pathogens exist in phenotypically distinct strains that interact with each other through competition for hosts. General models that describe such multi-strain systems are extremely difficult to analyze because their state spaces are enormously large. Reduced models have been proposed, but so far all of them necessarily allow for coinfections and require that immunity be mediated solely by reduced infectivity, a potentially problematic assumption. Here, we suggest a new state-space reduction approach that allows immunity to be mediated by either reduced infectivity or reduced susceptibility and that can naturally be used for models with or without coinfections. Our approach utilizes the general framework of status-based models. The cornerstone of our method is the introduction of immunity variables, which describe multi-strain systems more naturally than the traditional tracking of susceptible and infected hosts. Models expressed in this way can be approximated in a natural way by a truncation method that is akin to moment closure, allowing us to sharply reduce the size of the state space, and thus to consider models with many strains in a tractable manner. Applying our method to the phenomenon of antigenic drift in influenza A, we propose a potentially general mechanism that could constrain viral evolution to a one-dimensional manifold in a two-dimensional trait space. Our framework broadens the class of multi-strain systems that can be adequately described by reduced models. It permits computational, and even analytical, investigation and thus serves as a useful tool for understanding the evolution and ecology of multi-strain pathogens.

## Author Summary

Many important human pathogens, including HIV and influenza viruses, consist of many different “strains,” which elicit distinct immune responses in their hosts. Infection by one variant usually triggers partial cross-immunity against several other variants. This process leads to a complicated and dynamic immunity structure in the host population. Most existing models of a population with a multi-strain pathogen are either very complex, or rely on specific simplifying assumptions. Here, we suggest a new way of simplifying such models that allows for greater flexibility in underlying assumptions. This approach could lead to deeper understanding of the ecology and evolution of multi-strain pathogens. We apply our approach to a simple model of evolution of influenza A that illustrates one hypothesis about how influenza A evolution may be structured.

## Introduction

Microbial pathogens are tremendously diverse. Pathogens that cause one and the same disease may differ remarkably in both their genotype and their phenotype, like in HIV/AIDS [1], influenza [2], malaria [3], and meningitis [4]. Phenotypically different variants of the same pathogen are called strains. If several strains exist in a host population, they interact with each other in two ways.

The first type of interaction may be referred to as ecological interference [5,6]. For many infectious diseases, a host infected with one strain is removed, for the duration of the disease, from the population of hosts susceptible to the pathogen. This is because (a) the immune system of the host becomes activated upon infection by the first strain, so that it is hard for a second strain to enter and/or replicate in this host, and (b) the infected host may be physically removed from the susceptible population, by dying or staying at home. Ecological interference takes place even between unrelated pathogens [6].

The second type of interaction, referred to as cross-immunity interference, is specific to different strains of the same pathogen: these can confer full or partial immunity to each other. This means that a host infected with one strain becomes substantially less susceptible to certain other strains of the pathogen for a prolonged period of time after the initial infection is cleared. Cross-immunity is highest between phenotypically similar strains. Since phenotypic similarity usually implies recent common ancestry, a pathogen's ecology is thus intrinsically entangled with its evolution.

Understanding the dynamics of multi-strain pathogens at a general theoretical level turns out to be extremely difficult. Numerous models have been proposed during the past twenty years (e.g., [3,7–9]). Although these models share many similarities, they substantially differ in particulars, often resulting in conflicting model predictions. In consequence, there is little agreement as to how best to gain insights into the ecology and evolution of multi-strain pathogens. Models of multi-strain pathogens can be either equation- or agent-based. Agent- or individual-based models have recently become increasingly elaborate and interesting [10–13], largely due to an increase in computational capabilities. Since these models, however, are not designed for analytical tractability, we do not dwell on this type of model here.

Virtually all equation-based models of disease dynamics can be traced back to the compartment model introduced by Kermack and McKendrick in 1927 [14]. These models are also known as SIR (susceptible–infected–recovered) models, reflecting a host population's partitioning into susceptible, infected, and recovered individuals. A problem that arises immediately when attempting to extend this classical SIR framework to multiple strains is that the number of state variables, and typically also of parameters, increases exponentially with the number of strains [8,9]. This presents not only computational challenges but also draws attention to a fundamental conceptual difficulty: even for a moderately large number of strains, the resultant number of state variables quickly surpasses any realistic host population size. Most compartments in such a model therefore consist of few individuals, if they are occupied at all: effects of demographic stochasticity must then not be neglected. To avoid this complication, existing approaches to modeling multi-strain pathogens have attempted to reduce the number of model compartments. Usually, such reductions are valid only under certain sets of assumptions that may or may not be adequate depending on the modeled phenomenon. Thus, it is important to expand the set of assumptions under which reduced models are applicable. Our work presented here contributes to this goal.

Traditionally, full models have been developed based on the assumption of reduced susceptibility, which implies that immune hosts are able to block off an infection completely, with a certain probability [8,9]. On the other hand, all existing reduced models rely on the assumption of reduced infectivity that implies that all hosts, immune or not, get infected with the same probability, but those that possess immunity become less infectious than those who do not [3,15]. The reality, most likely, lies somewhere between these two abstractions. Nevertheless, as we discuss in the Model section, the reduced susceptibility assumption seems more plausible. In this study, we develop a state-space reduction approach that can be applied under either of these assumptions, in models with or without coinfections. Our approach differs from the existing ones in that it produces a collection of models that approximate the full models with the desired degree of accuracy. The number of variables needed for the resulting approximations grows algebraically with the number *n* of strains, rather than exponentially: when *n* is large, the difference between, e.g., *n* ^2^ and 2*^n^*, is enormous, with the former growing much more slowly than the latter. If coinfections and reduced infectivity are assumed, our approach produces a model equivalent to that of Gog and Grenfell [15].

To illustrate the utility of our approach, and that of reduced models in general, we demonstrate its application to the phenomenon of drift in influenza A. Using reduced models we are able to simulate up to 400 strains. Influenza A is a multi-strain pathogen whose epidemiology and evolution display an intricate interaction pattern. Because the human immune system can produce protective antibodies against influenza's surface glycoprotein hemagglutinin, individuals gain lifelong immunity against each strain of the virus with which they have been infected [2,16]. This results in a complex partitioning of the human host population according to the immunity of individuals to different influenza strains. The ensuing frequency-dependent selection is thought to drive the evolution of influenza A, giving rise to a process known as antigenic drift [17]. Lapedes and Farber [18] have shown that the antigenic space of influenza is approximately five-dimensional. Subsequently, Smith et al. [19] argued that the first two principal dimensions are most important. Moreover, as follows from results by Smith et al., the temporal evolution of influenza's H3N2 subtype proceeds along a single line in the antigenic space, i.e., antigenic clusters corresponding to different years are well separated along the first principal dimension. This agrees with the observation that the phylogenetic tree of subtype H3N2 possesses a single trunk [20] (but see an alternative hypothesis by Recker et al. [21]). In other words, even though the H3N2 subtype experiences substantial genetic diversity during each epidemic season, only one progeny strain survives in the longer run. Accordingly, the number of coexisting H3N2 strains does not grow from year to year.

A few recent studies have attempted to model, and thereby explain, the phenomenon of antigenic drift in influenza A. Apart from individual-based models, most of these studies consider a one-dimensional strain space in which some sort of traveling-wave behavior is observed [15,22–25]. To constrain the evolution of a virus to one dimension in a two-dimensional strain space, it has been necessary to require that the strain space was essentially unviable except for a relatively thin region along one axis [15].

In a recent study, Koelle et al. [26] took a different approach and succeeded in constraining the diversity of a virus living in a high-dimensional sequence space. The authors explicitly mapped viral genotypes to phenotypes and showed that the single-trunk phylogeny of influenza A may be a consequence of the neutral network structure of the influenza genotype space. However, it is an open question which properties of the phenotype space are sufficient to constrain viral diversity in the course of its evolution. Recker et al. [21] suggest one explanation. They argue that the succession of antigenically distinct variants may be an intrinsic feature of the dynamics of a limited set of antigenic types that are always present in the host population and, thus, is decoupled from the genetic evolution of the virus. Here, we suggest an alternative conceptual scenario that follows the more traditional view that antigenic drift and genetic evolution are tightly connected. However, we deliberately avoid the problem of mapping genotypes to phenotypes and, instead, assume a relatively simple structure of the phenotype space—a rectangular lattice. Our model offers a straightforward explanation of what could be happening in such a phenotype space in order for the diversity of a virus to be constrained in the long run. Our work is, thus, complementary to that of Koelle et al. [26]. In our two-dimensional phenotype space, each coordinate captures changes in the conformations of an epitope—local region on the surface of the hemagglutinin molecule that interacts with the immune system [27–29]. We then investigate a scenario in which the immune response of hosts depends on two epitopes, and full immune protection is gained against all strains sharing an epitopic conformation with a previous infection. In this respect, our model is closely related to models studied by Gupta and colleagues [3,21,30]. We show that the evolutionary trajectory of the influenza A virus in our model follows a line, even though the model's strain space is two-dimensional. This finding agrees with the observed single-trunk phylogeny of influenza's H3N2 subtype [20].

## Model

### Types of Equation-Based Multi-Strain SIR Models

As mentioned above, SIR models are based on partitioning a host population into susceptible, infected, and recovered classes. Abundances in the resultant compartments are then tracked through time by means of ordinary differential equations. There are three major dichotomies according to which multi-strain SIR models can be classified. The first dichotomy refers to the treatment of individuals with respect to cross-immunity: there are history-based and status-based approaches.

In the history-based approach introduced by Castillo-Chavez et al. [31] and generalized by Andreasen et al. [8], the hosts are grouped into classes by their disease histories, which are defined in terms of all strains with which an individual has ever been infected. Disease histories determine the rates at which these compartments are populated and depopulated when hosts acquire infections with new strains.

In the status-based approach introduced by Gog and Swinton [9], the individuals are grouped together by their immune status, which is defined in terms of all strains against which an individual is immune. This set is at least as large as the set of all strains with which an individual has been infected, provided one assumes that infection results in complete and persistent protection. By definition, an individual is fully susceptible to all strains not included in its immune status. The immune status determines the rate at which the compartment is populated and depopulated when individuals acquire immunity against new strains. In particular, after an infection with a new strain, individuals move to new immune status classes with rates that are determined by the probabilities of acquiring cross-immunity against other strains. Thus, after an infection with a particular strain, different individuals with the same immune status change their immune status in different ways. This approach to capturing the probabilistic aspect of cross-immunity is called polarized immunity.

The second dichotomy is the permission or prohibition of coinfections. Coinfection is an event through which an individual, while already being infected with one strain, gets simultaneously infected with a second strain. Unrestricted permission or complete prohibition of coinfections are, of course, mathematical abstractions. One or the other may be more plausible for any particular pathogen.

The third dichotomy refers to the way protective immunity works. As mentioned above, either the chance for an immune host to get infected or the infectivity of an immune host during a secondary infection is reduced. Within the history-based approach, models constructed under both assumptions were shown to behave qualitatively similar, at least in simple systems [32,33]. A mathematical formulation of a status-based multi-strain model usually forces the modeler to make a choice between the reduced-susceptibility assumption and the reduced-infectivity assumption. The latter is more convenient from the mathematical standpoint, as such models easily yield themselves to state-space reduction. This is because the rate at which hosts acquire infection and, hence, immunity is assumed to be independent of the immune status (or disease history, in history-based models). However, within the status-based framework, this assumption is somewhat problematic from the biological standpoint.

In general, infection of a host with a virus results in two effects: (a) the host becomes sick and transmits the virus, and (b) the immune system of the host develops protective antibodies against the infecting variant. If the host is already partially immune to the infecting strain, that is, protective antibodies had been developed prior to the infection, then (a) the severity and the duration of infection are reduced, and (b) the production of new types of antibodies is slowed down. In the limit case, when the host has full immune protection against the infecting strain, it is capable of completely fending off the infection with the existing arsenal of antibodies, resulting in no infectiousness and no production of new types of antibodies. In the simplest version of status-based models, individuals are either fully susceptible to a strain or fully immune against it, so this limit case should apply. The assumption of reduced infectivity within this framework correctly captures the first effect but neglects the second effect. In other words, hosts that are fully immune against a particular variant do not transmit it, but, paradoxically, still increase their repertoire of antibodies in exactly the same way as do susceptible individuals, upon an infection with this variant (see Protocol S1 for mathematical implications of this assumption). Evidently, this results in an over-immunization of the host population. In this light, the assumption of reduced susceptibility seems more plausible, at least within the status-based framework. In the remainder of this study, we will therefore work under this assumption, even though our approach is applicable to models with reduced infectivity as well.

Full history-based or status-based multi-strain SIR models are cumbersome in terms of their analytical treatment [33,34] and numerical analysis [35], mainly due to the enormous size of their state spaces. Therefore, reduced models are necessary and have been proposed in the past. However, as mentioned above, only models that allow for coinfections and work under the reduced-infectivity assumption yielded themselves so far to reduction [15,30,32], whereas models that prohibit coinfections and those that work under the assumption of reduced susceptibility remained intractable. With a view toward filling this gap within the status-based framework, we propose a new state-space reduction approach that is based on two key observations:

1. When attempting to infect a potential host, an infecting strain does not in any way “perceive” the host's entire immune status or disease history. What it does perceive is simply whether or not this potential host possesses any immunity against the focal strain. This consideration suggests a natural set of state variables: it is helpful to keep track of the proportions of a population that are immune to each strain, or combinations thereof. It turns out to be possible to reformulate any full status-based model in terms of such new immunity variables. Below we refer to this transformation as an “expansion in immunity variables.”

2. The utility of this transformation becomes clear when we recognize that, at any moment in time and for most diseases, many hosts will be immune to only a few of the strains currently circulating, while only a few hosts will be immune to many of these strains. Consequently, the immunity variables that describe the latter small proportions of the host population need not be tracked exactly and independently but can instead be approximated, without much disturbing the overall disease dynamics. Higher-order immunity variables can thus be approximated by functions of lower-order immunity variables. Below we refer to this approximation as truncating or “closing” the disease dynamics at a desired order.

The approach presented in this article introduces a general representation of status-based multi-strain models in terms of immunity variables. This representation is useful because it produces a hierarchical structure of equations describing the dynamics of a multi-strain system. The equations at any given order ℓ of this hierarchy are decoupled from equations at all orders above ℓ + 1, under the assumption of reduced susceptibility, or even above ℓ, under the assumption of reduced infectivity. Thus, this hierarchy can easily be truncated at any order, either by approximating higher-order immunity variables with functions of lower-order variables under the former assumption, or by simply ignoring higher-order variables under the latter assumption. The resultant truncated models provide either approximate or exact reduced descriptions of the original system.

### Model Description

We now proceed to the mathematical formulation of our framework. For the sake of clarity, we develop our reasoning for the model with coinfections; the model in which coinfections are prohibited is outlined in Protocol S1. The following notations are used throughout (see Table 1 for a full summary): *K* is the set of all *n* strains; individual strains from this set are referred to by their index *i*; *S_A_* is the proportion of individuals in the host population that possess immune status *A⊂K* and that, therefore, are currently fully susceptible to all strains in the subset *K*\*A* (we refer to these individuals as being *in state A*); *S*_∅︀_ thus denotes the class of naive individuals, i.e., individuals that have no immunity whatsoever; and *I_i_* is the proportion of individuals in the host population that are currently infectious with strain *i*. Since all host individuals naturally fall in exactly one of the *S_A_* classes, we have Σ*_A_*_⊂__*K*_ *S_A_* =1. Here, the summation is taken over all subsets of the set of strains, including the empty set.

**Table 1:**
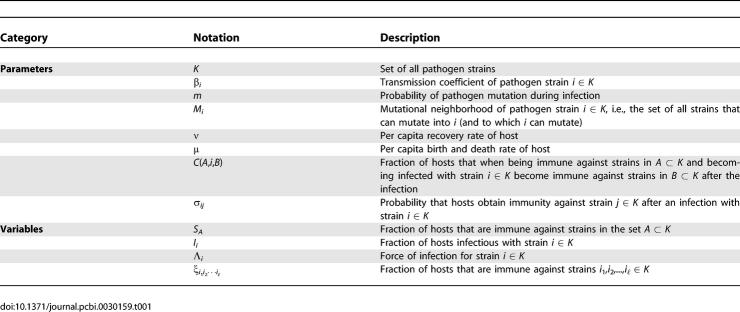
Notations Used Throughout This Study

Based on this notational framework, we can specify the disease dynamics of a multi-strain pathogen in three steps, (a) to (c) below, by considering the three processes that cause host individuals to enter and exit classes defined by their immune status.

(a) *Births and deaths.* We assume that hosts are born at per capita rate μ, being uninfected and susceptible to all strains. Accordingly, all newly born hosts enter the population through class *S*_∅︀_. The birth rate into class *S_A_* is thus given by μδ*_A_*_,∅︀_, where

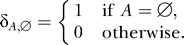



We further assume that infections do not alter the death rate of hosts and that the host population is at its demographic equilibrium. This implies a constant per capita death rate μ for all classes.

(b) *Acquisition of immunity.* The crucial ingredient in this type of model is to specify how an infection changes the immune status of the infected host. We assume that hosts recover from an infection at per capita rate ν. The proportion of hosts that recover to a state *B ⊂ K*, having been in state *A ⊂ K* when they were infected by strain *i* ∈ *K*, is denoted by *C*(*A*,*i*,*B*). Following Gog and Swinton [9], we assume that *C*(*A*,*i*,*B*) is allowed to take non-zero values only if: 1) *i* 蜓 *B*, which means that each strain confers total immunity to itself; 2) *A ⊂ B*, which means that immunity is only gained; 3) *i* 蜓 *A*, which means that, to get infected, hosts must be susceptible to the infecting strain.

Any chosen set of functions *C* also has to fulfill the consistency condition

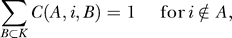
since there are no deaths from infection. Note that point 3 above is the mathematical formulation of the reduced-susceptibility assumption (compare the three assumptions to those of Gog and Grenfell [15] provided in Protocol S1).

(c) *Acquisition of infections.* The force of infection for strain *i*, describing the per capita rate at which host individuals susceptible to stain *i* get infected by strain *i*, is Λ*_i_* = β*_i_I_i_*, where β*_i_* is the transmission coefficient for strain *i*. Note that mutations between strains can be easily incorporated into this dynamics, by adjusting the force of infection,

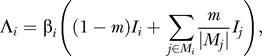
where *M_i_ ⊂ K* is the mutational neighborhood of strain *i*, i.e., the set of all strains that can mutate into *i*. Depending on the chosen description of the pathogen, *M_i_* can be, for example, the set of all point-mutation neighbors or a set of neighbors in a phenotype space. |*M_i_*| denotes the number of strains in this neighborhood. We assume that all neighborhoods are mutual, i.e., *i* ∈ *M_j_* if and only if *j* ∈ *M_i_* for any *i*, *j* ∈ *K*. We assume that mutations can occur with probability *m* during the infection period.

Based on (a) to (c), we obtain the following system of equations [9],

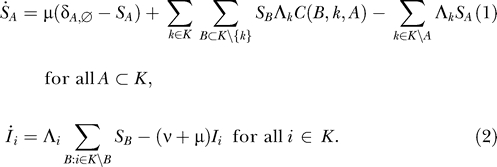
where dots denote derivatives with respect to time. Altogether, this system contains 2*^n^* + *n* − 1 equations, where *n* is the number of strains.

To complete the definition of our multi-strain model, we further specify the process of immunity acquisition. We introduce the probability 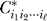 (*A*,*k*), ℓ = 1,2,…, *n* of acquiring immunity against strains *i*_1_,*i*_2_,…,*i*_ℓ_ (all different) after an infection with strain *k* for a host that had immune status *A* prior to the infection,

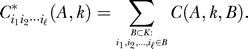



We assume that (a) the chance of obtaining immunity against strain *i* after the infection with strain *k* does not depend on the previous immune status of the individual, and (b) if strain *k* can potentially confer cross-immunity to strains *i* and *j*, then obtaining immunity against *i* and obtaining immunity against *j* are independent. According to (b), the chance that a host becomes immune to strains *i* and *j* after being infected with strain *k* is simply σ*_ki_*σ*_kj_*, where σ*_ki_* and σ*_kj_* are the chances of acquiring immunity against strains *i* and *j*, respectively. Mathematically, these assumptions determine the shape of the C^*^-functions defined above. All functions 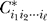 (*A*,*k*) vanish if *k* ∈ *A* and, if *k* 蜓 *A* we have

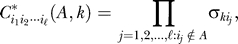
where, by definition, Π*_j_*_∈∅︀_ σ*_kj_* = 1. Based on these assumptions, cross-immunity is entirely characterized by the matrix of pairwise cross-immunity coefficients, σ = (σ*_ij_*), *i*,*j* ∈ *K* [9]. The elements of σ are all probabilities, so that σ*_ij_* ∈ [0,1] and σ*_ii_* = 1.

### Expansion in Immunity Variables

To derive the approximations of model (1)–(2), we rewrite that system in terms of the immunity variables,

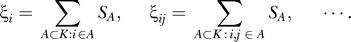



Each immunity variable has a clear intuitive interpretation: 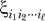 describes the proportion of hosts that currently have immunity against strains *i*_1_,*i*_2_,…,*i*_ℓ_ ∈ *K*. We will refer to immunity variables 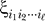 as being of order ℓ. Evidently, all immunity variables are symmetric with respect to the permutation of their indices. Immunity variables with duplicate indices *i* remain unchanged when the duplicate index is removed, ξ_…*i*…*i*…_ = ξ_…*i*…_. By definition, the immunity variables satisfy monotonicity conditions, 1 ≥ 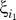 ≥ 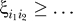 for all pairwise different *i*_1_,*i*_2_,…∈ *K*.

Recalling that Σ*_A_*_⊂__*K*_ *S_A_* = 1, we easily obtain the new equations for *İ_i_*,

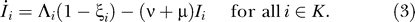



Derivation of the equations for ξ*_i_*, ξ*_ij_* etc., is more technical. Here we restrict ourselves to an explicit derivation of the equation for ξ*_i_*. For this purpose, we apply the time derivative to the definition of ξ*_i_* and use (1), to obtain

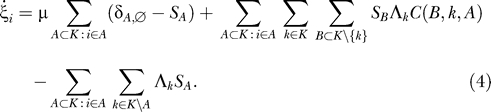



The first term in Equation 4 accounts for the depletion, due to deaths, of healthy host individuals that are immune to strain *i*,

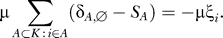



The second term in Equation 4 can be simplified as follows,

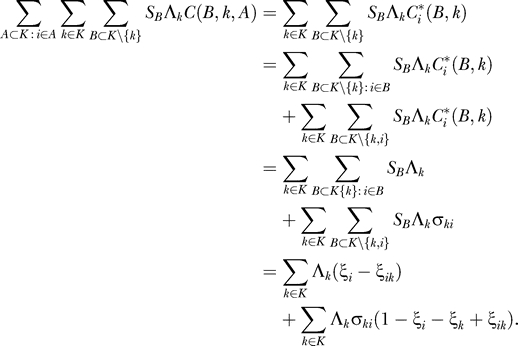



The last equality is satisfied due to the inclusion–exclusion principle [36]. Finally, the third term in Equation 4 can be rewritten as

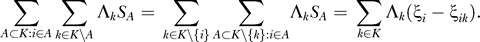



Collecting the three results above, we obtain the equation for ξ*_i_* expressed in terms of the new variables,

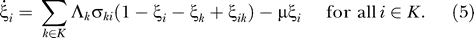



The equations for ξ*_i,j_* are obtained analogously,

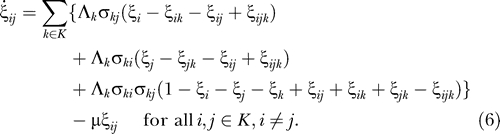



Fortunately, it is not necessary to rewrite the full system of Equations 1–2 in terms of immunity variables, since our goal is to reduce it. The equations for the immunity variables of order ℓ depend on the immunity variables of order ℓ and ℓ + 1, but not on any immunity variables of higher orders. To truncate this hierarchy of equations at order ℓ, we thus need to approximate the ξ-variables of order ℓ + 1 by a function of immunity variables of lower orders. This procedure is similar to moment-closure techniques widely used in, e.g., spatial ecology and epidemiology [37,38]. To distinguish the true ξ-variables from their approximations, we add a hat to the latter ones, as, e.g., in ξ̂*_ij_*. Each approximation must satisfy three conditions that directly follow from the definition of the ξ-variables:

1. Symmetry condition:

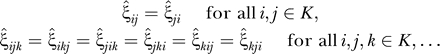



2. Monotonicity condition:

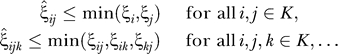



3. Redundancy condition: the approximate immunity variables with duplicate indices must be equal to the corresponding immunity variables of the previous order when the duplicate index is removed, 
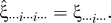


Below we introduce and discuss two simple closures of order 1 and one simple closure of order 2.

(a) *Order-1 independence closure:*

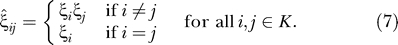



The symmetry and redundancy conditions are evidently met, and the monotonicity condition is satisfied because, by definition, ξ*_k_* ≤ 1 for all *k* ∈ *K*. The motivation underlying this closure is the following. If the cross-immunity interference between strains is small, and if the time that hosts spend in the infectious state is small compared with the time they spend in the healthy state, then hosts immune to each strain will be almost independently distributed among all hosts. We expect this closure to underestimate the level of immunity in the host population especially for high values of cross-immunity when correlations in the population immunity structure are high. However, even when cross-immunity between strains is absent altogether, the independence closure is not exact because of host aging (this can easily be checked for a simple system with two strains). It would be interesting to know whether there exists an exact closure in this special case.

(b) *Order-1 interpolation closure:*

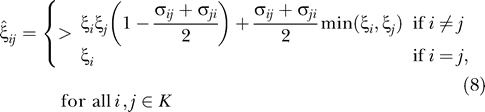



Again, the symmetry and redundancy conditions are evidently met, and the monotonicity condition is satisfied because ξ̂*_ij_* ≤ max{ξ*_i_*ξ*_j_*, min(ξ*_i_*,ξ*_j_*)}, and 0 ≤ (σ*_ij_* + σ*_ji_*)/2 ≤ 1 for all *i*,*j* ∈ *K*. If we rewrite this approximation for the case of a symmetric cross-immunity matrix σ,

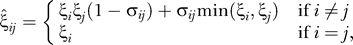
it is easy to see the motivation underlying this closure. It results from the linear interpolation between two extreme cases: absent cross-immunity and full cross-immunity. If, in the one extreme, σ*_ij_* = 0, the order-1 interpolation closure will coincide with the order-1 independence closure. If, in the other extreme, σ*_ij_* = 1, all hosts who have been infected with strain *i* (strain *j*) will be immune also to strain *j* (strain *i*). We approximate the fraction of hosts that have been infected with strain *i* (strain *j*) by ξ*_i_* (ξ*_j_*), which motivates the minimum term. This approximation is crude because we neglect cases in which immunity to strain *i* is mediated through a third strain *k* that may not provide cross-immunity to strain *j*.

(c) *Order-2 independence closure:*

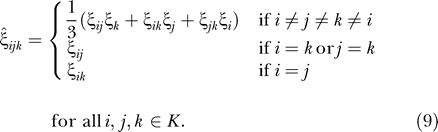



The motivation underlying this closure is analogous to that of the order-1 independence closure. All conditions are fulfilled.

Models truncated at first order have 2*n* remaining variables, while models truncated at second order have *n*(*n* + 3)/2 variables. Thus, our approximation has enabled a switch from an exponential to an algebraic scaling of the number of state variables with the number *n* of strains.

It is interesting to compare the obtained first-order equations under, say, the independence closure, with the reduced model proposed by Gog and Grenfell [15]. The latter is an exact reduced representation of the full status-based model with coinfections and reduced infectivity (we demonstrate this in Protocol S1), and, as such, can be used as an approximation for the full model with coinfections and reduced susceptibility. Gog and Grenfell's equations [15] are

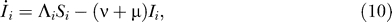


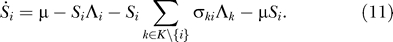



Now, we define the fraction of susceptibles to strain *i* as *S_i_* = 1 − ξ*_i_* and rewrite Equation 3, and Equation 5,

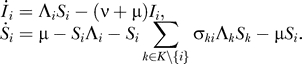



These equations are very similar to Equations 10 and 11, with the only difference occurring in the summation term in the equation for *Sƃ*_*i*_. As expected from the initial assumptions, immunization of the host population happens at a faster rate in Gog and Grenfell's model.

We have thus demonstrated how our framework of approximation—based on transformation to, and expansion in, immunity variables—provides simplified disease dynamics of multi-strain pathogens, in particular under the assumption of reduced susceptibility. In the next section we will compare, for a pathogen with four strains, the dynamics of the full system with that of the proposed approximations and Gog and Grenfell's model.

### Implementation Details

All numerical analyses were carried out using the MATLAB computing environment (The Mathworks, http://www.mathworks.com) with numerical accuracy 10^−7^. Code is available upon request.

## Results

### Comparison of Closures for a Pathogen with Four Strains

We consider a simple system of four strains along a line. The strain space is given by *K* = {1,2,3,4}, and strains adjacent on the line constitute the mutational neighborhood,

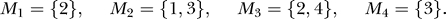



Adjacent strains also confer cross-immunity to each other, resulting in a tridiagonal cross-immunity matrix,

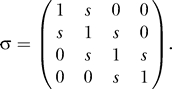



The functions *C*(*A*,*i*,*B*), describing the probability that a host's immune status changes from *A* to *B* owing to infection with strain *i*, are determined according to Gog and Swinton [9],

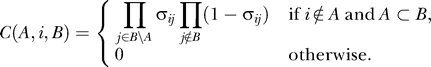



With such a small number of strains, the behavior of the full SIR model is tractable and can be used as a baseline reference. We can also examine how Gog and Grenfell's reduced infectivity model performs if we consider it as an approximation to the full model with reduced susceptibility. We numerically solve the following equations for the time interval [0,*T*] and for a range of parameters: 1) the full SIR system: Equations 1 and 2; 2) the Gog and Grenfell model: Equations 10 and 11; 3) the approximation based on the order-1 independence closure: Equations 3 and 5, where ξ*_ij_* are substituted according to Equation 7; 4) the approximation based on the order-1 interpolation closure: Equations 3 and 5, where ξ*_ij_* are substituted according to Equation 8; 5) the approximation based on the order-2 independence closure: Equations 3, 5, and 6, where ξ*_ijk_* are substituted according to Equation 9.

The following parameters are kept fixed: ν = 1, μ = 0, and *T* = 40. We choose β*_i_* = *R*_0_ for all *i*. We vary the transmission coefficient *R*_0_, the cross-immunity coefficient *s*, and the mutation probability *m* in the following ranges: *R*_0_ ∈ {2,3,4,5}, *s* ∈ {0,0.1,0.2,…1}, and *m* ∈ {10^−8^,10^−6^,10^−4^}. Initially, 99% of the host population is fully susceptible to all strains, while 1% is infected with strain 1, is immune against it, and is fully susceptible to all other strains.

To assess how well the reduced models approximate the full model, we introduce one qualitative and one quantitative accuracy measure. We consider an epidemic detection threshold ɛ = 10^−4^, describing the smallest proportion of infected hosts at which the disease can still be detected in the population. The results presented below are not particularly sensitive to the exact value of this parameter within three orders of magnitude (unpublished data). We denote by *f_i_*(*t*) the fraction of individuals infected with strain *i* as predicted by the full model, and by *g_i_*(*t*) the same fraction as predicted by one of the reduced models. We consider four situations. 1) Neither *f_i_*(*t*) nor *g_i_*(*t*) exceed the epidemic detection threshold within the considered time interval. In this case, we say that the approximation correctly captures the dynamics of the real system, both qualitatively and quantitatively. 2) Only *f_i_*(*t*) exceeds the epidemic detection threshold within the considered time interval, while *g_i_*(*t*) always stays below it. In this case, there is a qualitative difference in predictions, because the full model predicts an epidemic, while the reduced model does not. Then, if *Y_i_* ⊂ [0,*T*] is the set of moments in time at which *f_i_*(*t*) > ɛ, we define the qualitative accuracy measure as ρ*_i_* = *L*(*Y_i_*)/*T*, where *L*(*Y_i_*) is the sum of the lengths of time intervals during which such qualitative difference is observed for the *i*th strain; in other words, *L*(*Y_i_*) is the Lebesgue measure of the set *Y_i_*. 3) Only *g_i_*(*t*) exceeds the epidemic detection threshold within the considered time interval, while *f_i_*(*t*) always stays below it. In this case, again, there is a qualitative difference in predictions, because the reduced model predicts an epidemic, while the full model does not. Then, if *Y_i_* ⊂ [0,*T*] is the set of moments in time at which *g_i_*(*t*) > ɛ, we define the qualitative measure analogously to the second case, ρ*_i_* = *L*(*Y_i_*)/*T*. 4) Both *f_i_*(*t*) and *g_i_*(*t*) exceed the epidemic detection threshold within the considered time interval. In this case we say that there is no qualitative difference in the model predictions, but there still could be a quantitative difference between them. We quantify the latter by the absolute error of *g_i_*(*t*) with respect to *f_i_*(*t*),

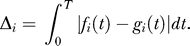



To obtain the overall qualitative error, ρ, we average ρ*_i_* over all strains, ρ = 1/4 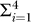 ρ*_i_*. This accuracy measure is suitable when we care most about determining which strains will cause epidemics and which will not, while ignoring quantitative differences in epidemic size and timing.

To obtain the overall quantitative accuracy measure, Δ, in a way that we could compare across models with different parameter values, we normalize Δ*_i_* by the total size of all epidemics, *Z* = 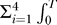 *f_i_*(*t*)*dt*, and sum over all strains, Δ = 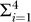 Δ*_i_*/*Z*. This accuracy measure is suitable when we care most about correctly capturing the shape and timing of major epidemics, while ignoring possible mistakes in minor epidemics.

**Figure 1:**
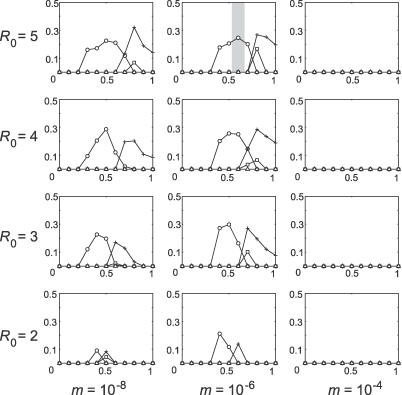
Qualitative Accuracy Measure ρ for the Performance of Four Models Qualitative accuracy measure (vertical axis) is shown in dependence on the cross-immunity coefficient *s* (horizontal axis), the transmission coefficient *R*_0_ (rows), and the mutation probability *m* (columns). Each panel shows the performance of four models: Gog and Grenfell's model (circles), order-1 independence closure (pluses), order-1 interpolation closure (squares), and order-2 independence closure (triangles). The highlighted region indicates the parameter combination *m* = 10^−6^, *R*_0_ = 5, and *s* = 0.6, for which the system's dynamics are shown in Figure 3.

**Figure 2:**
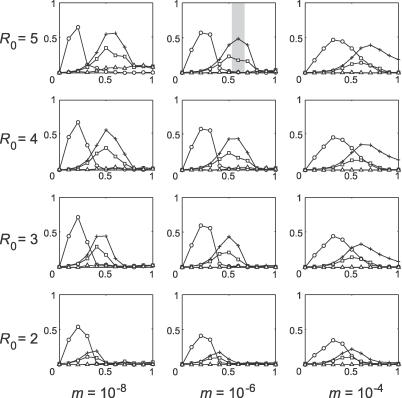
Quantitative Accuracy Measure Δ for the Performance of Four Models Details as in Figure 1.

**Figure 3:**
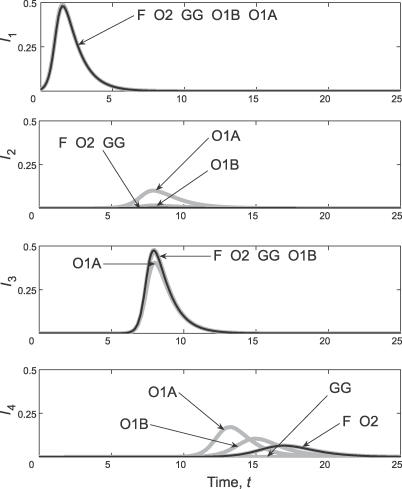
Dynamics of the Full Four-Strain SIR Model (Black Curves) Compared with Its Approximations (Thick Gray Curves) Solution of the full model, Gog and Grenfell's model, order-1 independence closure model, order-1 interpolation closure model, and order-2 independence closure model are denoted by F, GG, O1A, O1B, and O2, respectively. Parameter values: *m* = 10^−6^, *R*_0_ = 5, and *s* = 0.6.

The results of this comparison are shown in Figures 1–3. As could have been expected, the order-1 interpolation closure performs better than the order-1 independence closure, both qualitatively and quantitatively. The order-1 independence closure is quite accurate for small cross-immunity coefficients, but is problematic for medium and large cross-immunity coefficients. The order-1 interpolation closure, in turn, is most problematic for intermediate cross-immunity coefficients, which is again consistent with expectations. Indeed, owing to the nature of the interpolation that we used to determine ξ*_ij_*, we expect this closure to be more accurate for extreme values of cross-immunity and less accurate for intermediate values. As expected, the order-2 approximation performs substantially better than both order-1 approximations and Gog and Grenfell's model, making no qualitative errors at all and incurring quantitative errors on the order of a few percent. Gog and Grenfell's model is problematic for small and intermediate values of the cross-immunity coefficient, but, quite surprisingly, its error converges to zero for high values of cross-immunity where it becomes superior to the order-1 independence closure. This may have to do with the fact that the independence closure underestimates the level of cross-immunity of the population, especially for large values of cross-immunity.

We have conducted the same type of analysis for a circular four-strain system and for two six-strain systems (see Protocol S1)—the results turn out to be qualitatively similar to those presented here. Based on these numerical investigations, we conclude that the approximate model resulting from the order-1 interpolation closure offers a good compromise between accuracy and computational effort. The proposed order-2 approximation can be used if higher accuracy is required. Although Gog and Grenfell's model can also be used to approximate the dynamics of systems developed under the reduced-susceptibility assumption, if the degree of cross-protection between strains is high, we find that models developed under different assumptions lead, in general, to qualitatively different dynamics (see also Protocol S1). Despite the robustness of our conclusions on the quality of proposed closures with respect to key parameters within a sensible parameter region, further research is necessary to establish rigorous error bounds throughout the parameter space. In particular, we have not investigated the dependence of closure quality on the birth/mortality rate μ—performance may worsen for large μ. A more difficult, but equally important, question is how the topology of the strain space and of the cross-immunity structure alter the dynamics and the resultant performance of approximations in comparison with the full model.

### Application to Antigenic Drift in Influenza A

The application presented in this section illustrates how one could capture some aspects of the phenomenon of antigenic drift in influenza A's subtype H3N2 using models capable of tracing hundreds of variables. This is an attempt to extend the work by Gog and Grenfell [15] to the case of a two-dimensional viral phenotype space: those authors had shown that the dominant phenotype of the virus would move along a one-dimensional line in a two-dimensional strain space if it was assumed that viable phenotypes are concentrated in a narrow region along one of the axes. The structure of the influenza A phenotype space is not known, and it would be very surprising if all viable phenotypes were located only along a one-dimensional manifold. It is more likely that immuno-selection reduces the relative fitness of certain phenotypes at certain points in time [17]. Here, we construct an immunity-based mechanism that restricts the virus from explorg the whole phenotype space even if this space is mostly viable. We did not attempt to fit any parameters to real data, so that a quantitative correspondence, or even a close qualitative match, are not intended here.

We assume that the strain space of the considered influenza-like virus is a two-dimensional rectangular lattice, i.e., each variant of the virus is characterized by a pair of integers (*i*,*j*) ∈ *Z* ^2^ and the phenotypic mutational neighborhood is given by the next-neighbor relation on the lattice, *M*_(*i*,*j*)_ = {(*i* – 1,*j*),(*i* +1,*j*),(*i*,*j* – 1),(*i*,*j* + 1)}. While it is clear that the true strain space spanned by all viable conformations of the hemagglutinin protein does not resemble anything as simple as a two-dimensional rectangular lattice, by considering this example we still hope to shed some light on the mechanisms that govern the complicated evolutionary process of antigenic drift. This hope is not entirely unfounded. Plotkin et al. [39] have observed that changes in the influenza A hemagglutinin occur “along a different epitopic axis every 2–5 years.” Earlier, Wilson and Cox [29] noticed that amino acid substitutions in two epitopes on average were necessary to produce a new antigenic type. In addition to that, Smith et al. [19] have argued that the antigenic space of influenza A's subtype H3N2 is principally two-dimensional. Bringing these ideas together, we investigate influenza A evolution in an abstract two-dimensional phenotype space. We associate the movement along each dimension of this space with changes in states of a viral “effective epitope” (referred to as A and B below). The effective epitopes may not correspond to actual epitopes on the surface of the influenza A hemagglutinin protein (of which five are currently known) because not all epitopes may be immunogenic at all times. Below we drop the word “effective,” but it is always implied in the context of our model. In our interpretation, each pair of indices (*i*,*j*) ∈ *Z* ^2^ characterizing a particular strain implies that epitope A is in its *i*th conformation while epitope B is in its *j*th conformation.

**Figure 4:**
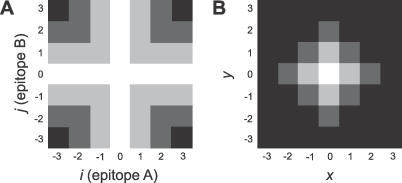
Cross-Immunity Structure Strains to which strain (0,0) confers full, 37%, 2%, or no cross-immunity are shown in white, light gray, dark gray, and black, respectively. (A) Our model. (B) Gog and Grenfell's model [15].

By the same token, it is natural to incorporate another idea, introduced by Gupta and colleagues [3,30]—the assumption that each individual that has been infected with strain (*i*,*j*) develops immunity against both epitopes, and thus gains full protection against all strains that possess the same conformation of either epitope. In other words, σ_(*i*,*j*)(*k*,ℓ)_ = 1 if either *i* = *k* or *j* = ℓ, resulting in a cross-shaped cross-immunity structure (Figure 4). The specificity of antibodies determines how fast cross-immunity decays when the epitopes of the challenging strain differ from the epitopes of the immunogenic strain. We follow [15] in choosing a Gaussian function for the decay of cross-immunity, with mean zero and standard deviation *a*, implying that *a* is the cumulative number of conformation changes in one epitope that reduces cross-immunity to *e* ^−1/2^ = 60.7%,

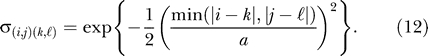



The cross-immunity matrix σ thus specified is sufficient for generating a one-dimensional trajectory of strain evolution: variants along the diagonal of strain space cause epidemics, whereas other variants do not (see Protocol S1). This conforms with intuition. Indeed, once a strain causes an epidemic, the host population acquires immunity not only against it but also against all the strains that share at least one epitope conformation with it, i.e., against all strains that have either the same *x*-coordinate or the same *y*-coordinate. Thus, the phenotypically closest mutant that can cause the next epidemic must differ from the current epidemic strain in both epitopes—so that this mutant can only be its diagonal neighbor.

**Figure 5:**
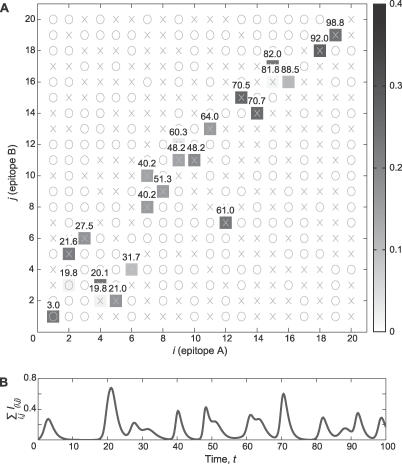
Approximate Dynamics of Antigenic Drift in Influenza A, Based on the Order-1 Interpolation Closure Parameter values: μ = 0, ν = 1, *m* = 10^−4^, and *a* = 1/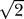 ; the heterogeneous transmission coefficients β_(*i*,*j*)_ were drawn from a normal distribution with mean 3 and standard deviation 0.5. The numerical solution for the time interval *t* ∈ [0,100] was obtained for a strain space given by a 20 × 20 rectangular lattice. The initial condition was given by all state variables being zero except for *I*_(1,1)_(0) = 0.01 and ξ_(1,1)_(0) = 0.01, corresponding to a healthy and fully susceptible host population with 1% of hosts infected with strain (1,1). (A) Strains whose maximum epidemic size exceeded 0.01 are shown. The gray shade indicates the maximum epidemic size; the number above each shaded square indicates the time when the maximum of the epidemic for that particular strain was reached. Circles indicate strains whose transmission coefficients are less than 3; crosses indicate strains with transmission coefficients greater than 3. (B) The sum of all proportions of infectious hosts as a function of time.

In this simple model, one strain strictly dominates the host population at each epidemic season. In a slightly more general setting, several strains can coexist within an epidemic season [19]. To account for this possibility, we introduce some heterogeneity in the transmission coefficients of strains. This is indeed as expected in reality, since some strains are likely to be slightly more virulent than others, because the conformation of the hemagglutinin protein influences how effectively a virus can penetrate target cells [28,29]. The amount of heterogeneity determines how many strains can coexist. In this version of our model, one to three strains usually coexist during an epidemic, while the principal component of the evolutionary trajectory remains one-dimensional (Figure 5). However, we conjecture that, if we considered an infinite rectangular lattice and started from the same initial condition, we would have observed either two lineages evolving in opposite directions along one of the two diagonals, or even four lineages evolving along both diagonals. We did not investigate this scenario in detail because it presumes that there is no immunity in the population whatsoever at the initial time point. This may be true for real influenza right after a reassortment event, but the evolutionary forces active during and right after reassortment are probably quite different from those governing the subsequent antigenic drift, and go far beyond the subject of our concern here. Thus, considering only the first quadrant of the phenotype space when starting viral evolution at the origin is equivalent to assuming that antigenic drift has already been going on for some time. The question thus addressed is how antigenic drift with limited diversity can be sustained.

To show that this result is a consequence of the immunity structure suggested here, rather than just a peculiarity of the considered equations, we have simulated exactly the same system for Gog and Grenfell's equations and for the model with no coinfections. We thus can show that the results reported above are qualitatively robust to model choice (see Protocol S1). Moreover, the assumptions of our model can be relaxed along two directions: (a) we may allow for mutations to more and more distant neighbors, with decreasing probabilities; and (b) cross-immunity may be allowed to decay slowly along the epitopic axes. As long as the immunity neighborhood extends farther along the epitopic axes than the mutational neighborhood, our qualitative conclusions hold (unpublished data). On the contrary, if the local cross-immunity structure suggested in [15] is used instead of the epitope-based one, viral evolution is no longer contained to a one-dimensional manifold (see Protocol S1).

The real influenza virus, which is likely to live in an approximately two-dimensional strain space, appears to experience a selection regime that, while allowing for the temporary coexistence of a small number of variants, constrains the long-term evolution of the virus to a single branch. Our model suggests a possible mechanism for explaining this surprising reduction. In particular, we conjecture that two ingredients are responsible for the associated evolutionary dynamics. 1) The first ingredient is the non-local nature of the immune response after an infection. This results from the fact that cross-immunity protects hosts not only against strains that are very similar to the infecting strain, but also against strains that are quite distant from it in strain space, as long as at least one of their epitope conformations resembles that of the infecting strain. Non-locality of the immune response prevents the virus from conquering the entire trait space. 2) The second ingredient, which enables temporary coexistence of several strains, is the heterogeneity of transmission coefficients in trait space. Paradoxically, this heterogeneity occasionally leads to temporary parity among the effective reproduction ratios of different strains. The effective reproduction ratio of a particular strain is the quantity that determines whether this strain takes off and causes an epidemic or dies out without ever reaching the epidemic threshold [40]. In our model, the effective reproduction ratio *R*_(*i*,*j*)_ of strain (*i*,*j*) equals β_(*i*,*j*)_ *S*_(*i*,*j*)_/ν. When all strains possess the same transmission coefficient, effective reproduction ratios thus depend only on the fractions of susceptible individuals. Clearly, the pool of susceptibles to the diagonal strains is larger than the pool of susceptibles to nearby off-diagonal strains, because past infections have induced low cross-immunity against the former and high cross-immunity against the latter. Hence, the diagonal strains successively cause epidemics, while the off-diagonal strains do not—accordingly, no polymorphism can emerge, not even in the short term. By contrast, in a trait space that is heterogeneous with respect to the transmission coefficient, relatively small values of *S*_(*i*,*j*)_ for off-diagonal strains may occasionally be compensated by high values of β_(*i*,*j*)_. In this manner, the effective reproduction ratios for off-diagonal strains may become comparable to those for diagonal strains. If, in addition, strains with comparable effective reproduction ratios start from comparable initial conditions, they reach epidemic values around the same time. This leads to short-term polymorphisms.

## Discussion

The approach introduced here enables systematic reductions in the complexity of status-based models of multi-strain pathogens. It is applicable to models with or without coinfections, with reduced susceptibility or reduced infectivity. If coinfections are allowed and reduced infectivity is assumed, our approach coincides with that of Gog and Grenfell [15]. The key is to depart from the traditional compartment models by adopting the viewpoint of the pathogen. This allows restating full status-based models in terms of immunity variables and truncating the hierarchy of resultant equations at the desired order. Overcoming the exponential explosion of state variables in traditional models of multi-strain pathogens, we have shown that the complexity of our approximation grows only algebraically, i.e., proportionally to powers of the number of strains in the system. Numerically solving the resulting approximate equations, we show that they mimic relatively well the dynamics of full systems, even when the first-order closures are used.

It would be interesting to perform a rigorous mathematical analysis of the behavior of our approximations and compare it with the behavior of the full system [9]. In theory, the behavior of the full model should be mimicked more and more accurately when the chain of equations for immunity variables is truncated at higher and higher orders. Indeed, when sufficiently many of them are satisfied exactly, one obtains the original full model expressed in terms of immunity variables.

In this work we presented a state-space reduction approach that applies only to the class of status-based models. We focused on this class of models for two reasons. Apart from easier mathematical treatment, there is an important conceptual difference that favors status-based models, at least under the reduced susceptibility assumption. Consider a situation when a host is repeatedly challenged with a strain. In the history-based approach, the probability for a host to acquire an infection remains the same across successive challenges. Thus, a host that has successfully used cross-reacting antibodies to repel one or more challenges from a particular pathogen is just as likely to be infected at the next challenge as is a host with the same infection history that has never seen this particular pathogen. To us, the status-based assumption—that, if antibodies fend off the first challenge, subsequent challenges will fail too—seems more realistic. Nevertheless, it would be interesting to know whether a state-space reduction approach similar to the one presented here could also be applied to history-based models.

Using our framework, we have investigated a potentially general mechanism for constraining viral evolution to one-dimensional manifolds when the underlying strain space is two-dimensional. Based on general knowledge about the antigenic space of the real influenza virus, we considered a hypothetical influenza-like virus whose phenotype space was given by a regular lattice with the same dimensionality as the number of principal components of the virus' antigenic space. We associate the movement along the axes of the resultant phenotype space with changes in the conformation of the viral “effective epitopes.” We based our analysis on two plausible qualitative assumptions: (a) during an infection, immunity is independently generated against all effective epitopes, and (b) immunity against one effective epitope suffices for full protection against viruses with similar epitopes. The resultant cross-shaped cross-immunity neighborhood drives the evolution of the virus along the diagonal of the phenotype space. This observation offers a conceptually simple approach to understanding single-trunk phylogenies of infectious pathogens.

Qualitatively different hypotheses for explaining the single-trunk phylogeny of influenza were introduced earlier on by Ferguson et al. [10] and, recently, by Koelle et al. [26]. Ferguson et al.'s hypothesis was later given a theoretical justification by Andreasen and Sasaki [24]. Ferguson et al. suggested that a presumed strain-independent short-lived form of cross-immunity could be the decisive factor for restricting the diversity of influenza strains. In their model, strains were described by genetic sequences and an ad hoc rule was considered to translate the genetic distance between strains into an antigenic distance. In reality, however, this translation must be expected to be (a) highly degenerate—the antigenic space has only two principal dimensions—and (b) highly nonlinear—small genetic changes will sometimes cause large antigenic changes and vice versa [19]. Failure to take into account these two factors results in an inflation in the dimensionality of the strain space of influenza, which, in turn, may lead to a necessity for introducing additional assumptions such as the existence of strain-independent short-lived cross-immunity. Koelle et al.'s recent work [26] is an excellent demonstration of this point. The authors explicitly modeled the highly nonlinear and highly degenerate mapping from genotypes to phenotypes and thus were able to obtain single-trunk phylogenies without the assumption of strain-independent cross-immunity. Their results constitute an important step toward understanding the evolution of influenza A. However, it still remains to be understood exactly how the influenza virus moves through the phenotype space in such a way that its diversity remains restricted. In particular, what are the crucial properties of the phenotype space's topology that allow for such a peculiar evolution? Our work outlines one possible set of such properties. Namely, if each independent direction of the antigenic space is associated with an effective epitope, and if the effective epitopes are immunogenically independent, then a one-dimensional trajectory of antigenic drift naturally emerges from the epitope-based cross-immunity structure. Our model is, of course, simplistic, and much more needs to be done to improve our understanding of the phenotype evolution of influenza. In particular, large-scale immunological experiments, as well as further studies of the protein folding process, will be necessary to uncover the actual topology of the influenza virus strain space, its cross-immunity structure, and the types of immunity that need to be considered.

## Supporting Information

Protocol S1 Supporting Information Text and Figures (661 KB PDF)

## Abbreviation

SIR: susceptible–infected–recovered
