# Supplementary material for: On State-Space Reduction in Multi-Strain Pathogen Models, with an Application to Antigenic Drift in Influenza A
Source: PLoS Comput Biol. 2007 Aug 17;3(8):e159. doi: 10.1371/journal.pcbi.0030159 (PMC1949840; doi:10.1371/journal.pcbi.0030159)
Supplement: Supplementary file 1 [file pcbi.0030159.sd001.pdf]

On state-space reduction in multi-strain pathogen models, with an application to antigenic drift in influenza A

Sergey Kryazhimskiy*, Ulf Dieckmann,
Simon A. Levin, Jonathan Dushoff

Supporting information

This PDF file includes:
Supporting information text
Figures S1 – S19

*To whom correspondence should be addressed. E-mail: skryazhi@princeton.edu

This supporting information document contains four sections. The first section provides a derivation of the expansion in immunity variables of the model with coinfections and reduced infectivity and demonstates its connection to the reduced model presented in [S1]. In the second section we elucidate the expansion in immunity variables for a model without coinfections. In the third section we present an extended analysis of accuracy for Gog and Grenfell's model [S1], as well as for order-1 and order-2 closures in models with and without coinfections. The last section contains an additional analysis of the influenza A drift model.

State space reduction in a model with coinfections and reduced infectivity

In this section we derive the expansion in immunity variables for the full status-based model with coinfections and reduced infectivity and show that the truncation of this expansion at the first order leads to the model studied by Gog and Grenfell [S1]. Our approach provides a generalization of that model since immunity variables allow us to truncate the chain of equations at higher orders if a more detailed description of the immunity structure of the population is desired.

The general status-based model with coinfection was formulated by Gog and Swinton [S2]. However, it was constructed under the assumption of reduced susceptibility. The assumption of reduced infectivity enters in the definition of the function $C(A, i, B)$ that determines the proportion of the host population that, after an infection with strain i, changes its immune status from A to B, as well as in the properties of the function $C_i^*(A, k)$ (see below). The reduced infectivity assumption implies that $C(A, i, B)$ is allowed to take non-zero values only if:

1. $i \in B$;

2. $A \subseteq B$.

The difference between this and the assumptions on C made in [S2] is that $C(A, i, B)$ can take non-zero values if $i \in A$. Biologically, this implies that individuals are allowed to change their immune status due to a mere exposure to a strain even if this strain is already included in their immune status (see also the discussion in the main text). Mathematically, this assumption implies that the equations for the class S_A are different from equations (1) in the main text of our paper in that the second sum is taken over all subsets of the set of strains and the last sum is taken over all strains. Equations for I_i remain the same.

$$\dot{S}_A = \mu(\delta_{A,\emptyset} - S_A) + \sum_{k \in K} \sum_{B \subset K} S_B \Lambda_k C(B, k, A) - \sum_{k \in K} \Lambda_k S_A, \quad \text{for all } A \subset K, \quad \text{(S1)}$$

$$\dot{I}_i = \Lambda_i \sum_{B : i \in K \setminus B} S_B - (\nu + \mu) I_i, \quad \text{for all } i \in K. \quad \text{(S2)}$$

Analogously to the derivation in the main text, we define the probability $C^*_{i_1 i_2 \cdots i_\ell}(A,k)$ of obtaining immunity against strains $i_1, i_2, \ldots, i_\ell$ (all different) after an infection with strain k for a host that had immune status A prior to the infection. But now, according to the assumption of reduced infectivity, we assume that

$$C^*_{i_1 i_2 \cdots i_\ell}(A,k) = \prod_{\substack{j=1,2,\ldots,\ell: \\ i_j \notin A \cup \{k\}}} \sigma_{k i_j}.$$

This implies that (a) the chance of obtaining immunity against strain i after the infection with the strain k does not depend on the presence or absence of immunity against other strains, and (b) if the host is already immune to strain k (i.e., $k \in A$), the chance of getting immunity to strain i through cross-protection betwen strains k and i, is proportional to σ_{ki}. This is a biologically subtle point of the reduced-infectivity assumption (see the discussion in the main text).

Analogously to the derivation in the main text, to obtain the reduced version of the model (S1)–(S2), we introduce the immunity variables

$$\xi_i = \sum_{A: i \in A} S_A, \quad \xi_{ij} = \sum_{\substack{A \subset K: \\ i,j \in A}} S_A, \quad \ldots,$$

in terms of which we rewrite the system (S1)–(S2). The equation for I_i is transformed straightforwardly once one recalls that $\sum_{A \subset K} S_A = 1$.

$$\dot{I}_i = \Lambda_i \left(\sum_{B \subset K} S_B - \sum_{B: i \in B} S_B \right) - (\nu + \mu) I_i = \Lambda_i (1 - \xi_i) - (\nu + \mu) I_i. \tag{S3}$$

Differentiating the definition of ξ_i with respect to time and using (S1), we obtain

$$\dot{\xi}_i = \mu \sum_{A: i \in A} (\delta_{A,\emptyset} - S_A) + \sum_{A: i \in A} \sum_{k,B} S_B \Lambda_k C(B,k,A) - \sum_{A: i \in A} \sum_{k \in K} \Lambda_k S_A, \tag{S4}$$

We shall consider the three sums one after another. The first sum yields

$$\sum_{A: i \in A} (\delta_{A,\emptyset} - S_A) = -\xi_i$$

since the sum of $\delta_{A,\emptyset}$-terms gives zero. The second term can be transformed in the following way,

$$\begin{aligned} \sum_{A: i \in A} \sum_{k,B} S_B \Lambda_k C(B,k,A) &= \sum_k \sum_B S_B \Lambda_k C^*_i(B,k) \\ = \sum_{k \in K} &\left(\sum_{B: i \in B} S_B \Lambda_k C^*_i(B,k) + \sum_{B: i \in K \setminus B} S_B \Lambda_k C^*_i(B,k) \right) \end{aligned}$$

$$= \sum_{k\in K}\left(\sum_{B:i\in B} S_B\Lambda_k + \sum_{B:i\in K\setminus B} S_B\Lambda_k\sigma_{ki}\right)$$
$$= \sum_{k\in K}(\xi_i\Lambda_k + (1-\xi_i)\Lambda_k\sigma_{ki})$$

The transformation of the last sum in (S4) is also straightforward:

$$\sum_{A:i\in A}\sum_{k\in K}\Lambda_k S_A = \sum_{k\in K}\Lambda_k\xi_i.$$

Gathering all the terms above, we obtain:

$$\dot{\xi}_i = \sum_{k\in K}\Lambda_k\sigma_{ki}(1-\xi_i) - \mu\xi_i. \tag{S5}$$

The equation for $\dot{\xi}_{ij}$ is obtained analogously,

$$\begin{aligned}\dot{\xi}_{ij} &= \sum_{k\in K}\Lambda_k\left[\sigma_{ki}(\xi_j-\xi_{ij}) + \sigma_{kj}(\xi_i-\xi_{ij}) + \sigma_{ki}\sigma_{kj}(1-\xi_i-\xi_j+\xi_{ij})\right] - \\ &- \mu\xi_{ij} \quad \text{for all } i,j\in K,\ i\neq j. \end{aligned} \tag{S6}$$

Continuing this chain, we would obtain the full system (S1)–(S2) expressed in terms of immunity variables. Note, however, that equations for immunity variables of any particular order are uncopuled from equations for immunity variables of higher orders. Therefore, the chain of equations truncated at a particular order would exactly represent the dynamics of the immunity variables up to that order. In particular, if we truncate the chain of equations at order 1, we can express (S3), (S5) in terms of I_i and $S_i = 1-\xi_i$. This leads to the model studied by Gog and Grenfell [S1].

$$\begin{aligned}\dot{S}_i &= \mu(1-S_i) - \sum_{k\in K}\Lambda_k\sigma_{ki}S_i,\\ \dot{I}_i &= \Lambda_i S_i - (\nu+\mu)I_i,\end{aligned}$$

for all $i\in K$.

State space reduction in models with no coinfections

In this section we derive the expansion in immunity variables for a model with no coinfections under the reduced-susceptibility assumption. At the end of the section we also provide, without a derivation, the expansion in immunity variables for the model with no coinfections and reduced infectivity. We start out from a model with no coinfection that is analogous to the model with coinfections considered by Gog and Swinton [S2]. Here, we have to slightly change the meaning of our notations. S_A represents the proportion of

currently *non-infected* hosts that possess immune status $A \subset K$ and that, therefore, are currently fully susceptible to all strains in the subset $K \backslash A$ (we refer to these individuals as being *in state* A); and I_A^i is the proportion of individuals in the host population that are currently infected with strain i and had immune status $A \subset K$ before the current infection (which implies $i \notin A$). Since all host individuals naturally fall in exactly one of these classes, we have

$$\sum_{A \subset K} S_A + \sum_{i \in K} \sum_{A \subset K \backslash \{i\}} I_A^i = 1. \tag{S7}$$

The proportion of hosts that recover to state $B \subset K$, having been in state $A \subset K$ when they were infected by strain $i \in K$, is given by $C(A, i, B)$ that has the properties described in the main text. The following is the full system of equations for the model with no coinfections.

$$\begin{aligned} \dot{S}_A &= \mu(\delta_{A,\emptyset} - S_A) + \nu \sum_{k \in K} \sum_{B \subset K \backslash \{k\}} I_B^k C(B, k, A) \\ &- \sum_{k \in K \backslash A} \Lambda_k S_A, \quad \text{for all } A \subset K, \end{aligned} \tag{S8}$$

$$\dot{I}_A^i = \Lambda_i S_A - (\nu + \mu) I_A^i, \quad \text{for all } i \in K \text{ and } A \subset K \backslash \{i\}, \tag{S9}$$

where

$$\Lambda_i = \beta_i \left((1 - m) \sum_{A \in K \backslash \{i\}} I_A^i + \sum_{j \in M_i} \sum_{A \in K \backslash \{j\}} \frac{m}{|M_j|} I_A^j \right). \tag{S10}$$

Altogether, this system consists of $2^n + n2^{n-1} - 1$ equations, where n is the number of strains.

We now rewrite this system in terms of the proportions of hosts infected with strain i,

$$I_i = \sum_{A \subset K \backslash \{i\}} I_A^i,$$

and the immunity variables,

$$\xi_i = \sum_{\substack{A \subset K: \\ i \in A}} S_A, \quad \xi_{ij} = \sum_{\substack{A \subset K: \\ i,j \in A}} S_A, \quad \ldots,$$

$$\eta_i^k = \sum_{\substack{A \subset K \backslash \{k\}: \\ i \in A}} I_A^k, \quad \eta_{ij}^k = \sum_{\substack{A \subset K \backslash \{k\}: \\ i,j \in A}} I_A^k, \quad \ldots$$

where $\xi_{i_1 i_2 \cdots i_\ell}$ describes the proportion of hosts that are currently not infected and have immunity against strains $i_1, i_2, \ldots, i_\ell \in K$; and $\eta_{i_1 i_2 \cdots i_\ell}^k$ describes the proportion of hosts that are currently infected with strain k and have immunity against strains $i_1, i_2, \ldots, i_\ell \in K$. We will refer to immunity variables $\xi_{i_1 i_2 \cdots i_\ell}$ and $\eta_{i_1 i_2 \cdots i_\ell}^k$ as being of order ℓ. As in the model with coinfections, all immunity variables are symmetric with respect to the permutation of

their subindices. Immunity variables with duplicate subindices i remain unchanged when the duplicate subindex is removed, $\xi_{\cdots i \cdots i \cdots} = \xi_{\cdots i \cdots\cdots}$ and $\eta^k_{\cdots i \cdots i \cdots} = \eta^k_{\cdots i \cdots\cdots}$. When a strain index k appears in both an η-variable's superscript and subscript, the η-variable must be zero, $\eta^k_{\cdots k \cdots} = 0$. By definition, the immunity variables satisfy monotonicity conditions,

$$1 \geq H \geq \xi_{i_1} \geq \xi_{i_1 i_2} \geq \dots$$

and

$$1 \geq I_k \geq \eta^k_{i_1} \geq \eta^k_{i_1 i_2} \geq \dots$$

for all pairwise different $k, i_1, i_2, \ldots \in K$. Here $H = 1 - \sum_{i \in K} I_i$ is the proportion of healthy (i.e., not infected) host individuals.

Recalling that $\sum_{A \subset K} S_A + \sum_{i \in K} I_i = 1$, we easily obtain the equations for $\dot{I}_i$, $\dot{\eta}^k_i$, $\dot{\eta}^k_{ij}$ etc.,

$$\begin{aligned}
\dot{I}_i &= \Lambda_i S_i - (\nu + \mu) I_i \quad \text{for all } i \in K, & \text{(S11)}\\
\dot{\eta}^k_i &= \Lambda_k(\xi_i - \xi_{ik}) - (\nu + \mu)\eta^k_i \quad \text{for all } i, k \in K;\ i \neq k, & \text{(S12)}\\
\dot{\eta}^k_{ij} &= \Lambda_k(\xi_{ij} - \xi_{ijk}) - (\nu + \mu)\eta^k_{ij} \quad \text{for all } i, j, k \in K;\ i, j, k \text{ pairwise different}, & \text{(S13)}\\
&\cdots
\end{aligned}$$

where $S_i = H - \xi_i$ is the fraction of individuals that are currently healthy and susceptible to strain i. The force of infection for strain i, expressed in terms of new variables, is

$$\Lambda_i = \beta_i \left((1 - m) I_i + \sum_{j \in M_i} \frac{m}{|M_j|} I_j \right). \tag{S14}$$

The derivation of the equations for $\dot{\xi}_i$, $\dot{\xi}_{ij}$ etc. is more technical. Here, we restrict ourselves to an explicit derivation of the equation for $\dot{\xi}_i$. For this purpose, we apply the time derivative to the definition of ξ_i and use (S8), to obtain

$$\dot{\xi}_i = \mu \sum_{\substack{A \subset K: \\ i \in A}} (\delta_{A,\emptyset} - S_A) + \nu \sum_{\substack{A \subset K: \\ i \in A}} \sum_{k \in K} \sum_{B \subset K \setminus \{k\}} I^k_B C(B, k, A) - \sum_{\substack{A \subset K: \\ i \in A}} \sum_{k \in K \setminus A} \Lambda_k S_A. \tag{S15}$$

The first term in (S15) accounts for the depletion, due to deaths, of healthy host individuals that are immune to strain i,

$$\mu \sum_{\substack{A \subset K: \\ i \in A}} (\delta_{A,\emptyset} - S_A) = -\mu \xi_i.$$

The second term in (S15) can be simplified as follows,

$$\begin{aligned}
\sum_{\substack{A \subset K: \\ i \in A}} \sum_{k \in K} \sum_{B \subset K \setminus \{k\}} I^k_B C(B, k, A) &= \sum_{k \in K} \sum_{B \subset K \setminus \{k\}} I^k_B C^*_i(B, k)\\
= \sum_{k \in K} \sum_{\substack{B \subset K \setminus \{k\}: \\ i \in B}} I^k_B C^*_i(B, k) &+ \sum_{k \in K} \sum_{B \subset K \setminus \{k, i\}} I^k_B C^*_i(B, k)\\
= \sum_{k \in K} \sum_{\substack{B \subset K \setminus \{k\}: \\ i \in B}} I^k_B &+ \sum_{k \in K} \sum_{B \subset K \setminus \{i, k\}} I^k_B \sigma_{ki} = \sum_{k \in K} \eta^k_i + \sum_{k \in K} \sigma_{ki}(I_k - \eta^k_i).
\end{aligned}$$

The last equality is satisfied because

$$\sum_{k\in K}\sum_{B\subset K\setminus\{i,k\}} I_B^k\sigma_{ki} = \sum_{k\in K}\sigma_{ki}\left(\sum_{B\subset K\setminus\{k\}} I_B^k - \sum_{\substack{B\subset K\setminus\{k\}:\\ i\in B}} I_B^k\right).$$

Note that the second term in (S15) can be rewritten as

$$\sum_{k\in K}\eta_i^k + \sum_{k\in K\setminus\{i\}}\sigma_{ki}(I_k-\eta_i^k) + I_i,$$

showing that it accounts for the replenishment of healthy host individuals that are immune to strain i due to (a) recovery of hosts that have previously been immune to strain i, but have been infected with some other strain k; (b) recovery of hosts that, after having been infected with some strain k, have gained cross-immunity to strain i; and (c) recovery of hosts that have been infected with strain i. Finally, the third term in (S15) accounts for the infection of individuals, immune to strain i, with some other strain k, and can be rewritten as

$$\sum_{\substack{A\subset K:\\ i\in A}}\sum_{k\in K\setminus A}\Lambda_k S_A = \sum_{k\in K\setminus\{i\}}\sum_{\substack{A\subset K\setminus\{k\}:\\ i\in A}}\Lambda_k S_A = \sum_{k\in K}\Lambda_k(\xi_i-\xi_{ik}).$$

Collecting the three results above, we obtain the equation for $\dot{\xi}_i$ expressed in terms of new variables,

$$\dot{\xi}_i = \sum_{k\in K}\left[\nu\left(\eta_i^k+\sigma_{ki}(I_k-\eta_i^k)\right)-\Lambda_k(\xi_i-\xi_{ik})\right]-\mu\xi_i \quad \text{for all } i\in K. \tag{S16}$$

The equation for $\dot{\xi}_{ij}$ is obtained analogously,

$$\begin{aligned}\dot{\xi}_{ij} \;&=\; \sum_{k\in K}\Big[\nu\left(\eta_{ij}^k+\sigma_{ki}(\eta_j^k-\eta_{ij}^k)+\sigma_{kj}(\eta_i^k-\eta_{ij}^k)+\sigma_{ki}\sigma_{kj}(I_k-\eta_j^k-\eta_i^k+\eta_{ij}^k)\right)-\\ &-\;\Lambda_k(\xi_{ij}-\xi_{ijk})\Big]-\mu\xi_{ij} \quad \text{for all } i,j\in K,\ i\neq j.\end{aligned} \tag{S17}$$

Observe that the equations for the immunity variables of order ℓ depend on the η-variables of order ℓ and on the ξ-variables of order $\ell+1$, but not on any immunity variables of higher orders. To truncate this hierarchy of equations at order ℓ, in full analogy to the model with coinfections, we need to approximate the ξ-variables of order $\ell+1$ by a function of immunity variables of lower orders. Each approximation must, again, satisfy the symmetry, monotonicity and redundancy conditions outlined in the main text.

We suggest the following simple closures:

(a) *Order-1 independence closure:*

$$\hat{\xi}_{ij} = \begin{cases}\dfrac{\xi_i\xi_j}{H} & \text{if } i\neq j\\ \xi_i & \text{if } i=j\end{cases} \qquad \text{for all } i,j\in K. \tag{S18}$$

(b) *Order-1 interpolation closure:*

$$\hat{\xi}_{ij} = \frac{\xi_i \xi_j}{H}\left(1 - \frac{\sigma_{ij} + \sigma_{ji}}{2}\right) + \frac{\sigma_{ij} + \sigma_{ji}}{2}\min(\xi_i, \xi_j) \quad \text{for all } i, j \in K. \tag{S19}$$

(c) *Order-2 independence closure:*

$$\hat{\xi}_{ijk} = \begin{cases} \frac{1}{3H}\left(\xi_{ij}\xi_k + \xi_{ik}\xi_j + \xi_{jk}\xi_i\right) & \text{if } i \neq j \neq k \neq i \\ \xi_{ij} & \text{if } i = k \text{ or } j = k \\ \xi_{ik} & \text{if } i = j \end{cases} \quad \text{for all } i, j, k \in K. \tag{S20}$$

Models truncated at first order have $n(n+1)$ remaining variables, while models truncated at second order have $n(n^2+3)/2$ variables.

Finally, we provide, without a derivation, the reduced version of the model with coinfections under the assumption of reduced infectivity. First, the full model with this assumption differs from the model with reduced susceptibility in that the class I_A^i, where $i \in A$, is no longer empty, since we assume that hosts can be infected with a variant even if it is in their immune status. Such individuals will not, however, contribute to infectivity with strain i and, therefore, the force of infection is still given by expression (S10). Equation (S7) is transformed to $\sum_{A\subset K} S_A + \sum_{i\in K}\sum_{A\subset K} I_A^i = 1$, and instead of (S8)–(S9) we have

$$\dot{S}_A = \mu(\delta_{A,\emptyset} - S_A) + \nu \sum_{k\in K}\sum_{B\subset K} I_B^k C(B,k,A) - \sum_{k\in K}\Lambda_k S_A, \quad \text{for all } A \subset K, \tag{S21}$$

$$\dot{I}_A^i = \Lambda_i S_A - (\nu + \mu) I_A^i, \quad \text{for all } i \in K \text{ and } A \subset K. \tag{S22}$$

To obtain the reduced version of this model, we rewrite (S21)–(S22) in terms of

$$I_i = \sum_{A\subset K} I_A^i,$$

$$\xi_i = \sum_{\substack{A\subset K:\\ i\in A}} S_A, \quad \xi_{ij} = \sum_{\substack{A\subset K:\\ i,j\in A}} S_A, \quad \ldots,$$

$$\eta_i^k = \sum_{\substack{A\subset K:\\ i\in A}} I_A^k, \quad \eta_{ij}^k = \sum_{\substack{A\subset K:\\ i,j\in A}} I_A^k, \quad \ldots.$$

It can be shown that system (S21)–(S22) is equivalent to the following system.

$$\begin{aligned} \dot{I}_i &= \Lambda_i H - (\nu + \mu) I_i \\ \dot{\eta}_i^k &= \Lambda_k \xi_i - (\nu + \mu)\eta_i^k, \\ \dot{\eta}_{ij}^k &= \Lambda_k \xi_{ij} - (\nu + \mu)\eta_{ij}^k, \\ \dot{\xi}_i &= \sum_{k\in K}\left[\nu\left(\eta_i^k + \sigma_{ki}(I_k - \eta_i^k)\right) - \Lambda_k \xi_i\right] - \mu \xi_i \end{aligned}$$

$$
\begin{aligned}
\dot{\xi}_{ij} &= \sum_{k\in K}\Big[\nu\left(\eta^k_{ij} + \sigma_{ki}(\eta^k_j - \eta^k_{ij}) + \sigma_{kj}(\eta^k_i - \eta^k_{ij}) + \sigma_{ki}\sigma_{kj}(I_k - \eta^k_i - \eta^k_j + \eta^k_{ij})\right) - \\
&- \Lambda_k \xi_{ij}\Big] - \mu\xi_{ij} \\
&\vdots
\end{aligned}
$$

for all $i, j, k \in K,\ i \neq j$, where $H = 1 - \sum_{k\in K} I_k$ and Λ_i is defined by (S14). As it is common for models with reduced infectivity, the dynamics of the immunity variables of a particular order does not depend on the immunity variables of higher orders. Therefore, the truncation at a given order reproduces the true behavior of the immunity variables up to that order.

Extended analysis of accuracy for reduced models

In this section we present the results of the same type of analysis as in the main text, for additional strain space topologies and for the model with no coinfections. We use the following topologies:

Topology 1. Linear four-strain system as described in the main text.

Topology 2. Circular four-strain system. The mutational neighborhood and the cross-immunity matrix are given by

$$
M_1 = \{2,4\}, \quad M_2 = \{1,3\}, \quad M_3 = \{2,4\}, \quad M_4 = \{3,1\},
$$

$$
\sigma = \begin{pmatrix} 1 & s & 0 & s \\ s & 1 & s & 0 \\ 0 & s & 1 & s \\ s & 0 & s & 1 \end{pmatrix}.
$$

Topology 3. Linear six-strain system. The mutational neighborhood and the cross-immunity matrix are given by

$$
M_1 = \{2\}, \quad M_2 = \{1,3\}, \quad M_3 = \{2,4\}, \quad M_4 = \{3,5\}, \quad M_5 = \{4,6\}, \quad M_6 = \{5\},
$$

$$
\sigma = \begin{pmatrix} 1 & s & 0 & 0 & 0 & 0 \\ s & 1 & s & 0 & 0 & 0 \\ 0 & s & 1 & s & 0 & 0 \\ 0 & 0 & s & 1 & s & 0 \\ 0 & 0 & 0 & s & 1 & s \\ 0 & 0 & 0 & 0 & s & 1 \end{pmatrix}.
$$

Topology 4. Torus-like six-strain system (Figure S1). The mutational neighborhood and the cross-immunity matrix are given by

$$
\begin{aligned}
M_1 &= \{2,3,4\}, \quad M_2 = \{1,3\}, \quad M_3 = \{1,2,6\}, \\
M_4 &= \{1,5,6\}, \quad M_5 = \{4,6\}, \quad M_6 = \{3,4,5\},
\end{aligned}
$$

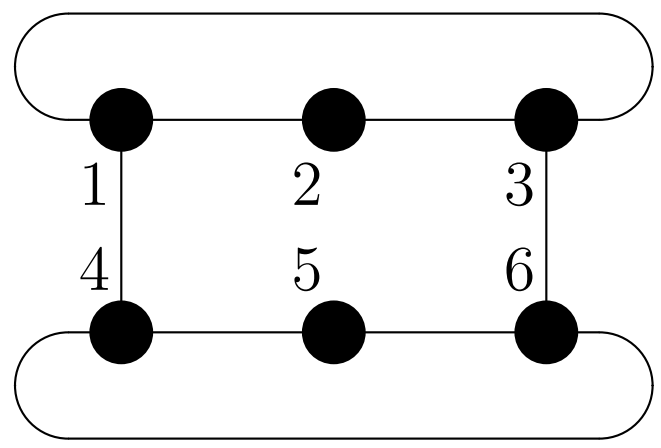

Figure S1: Topology 4.

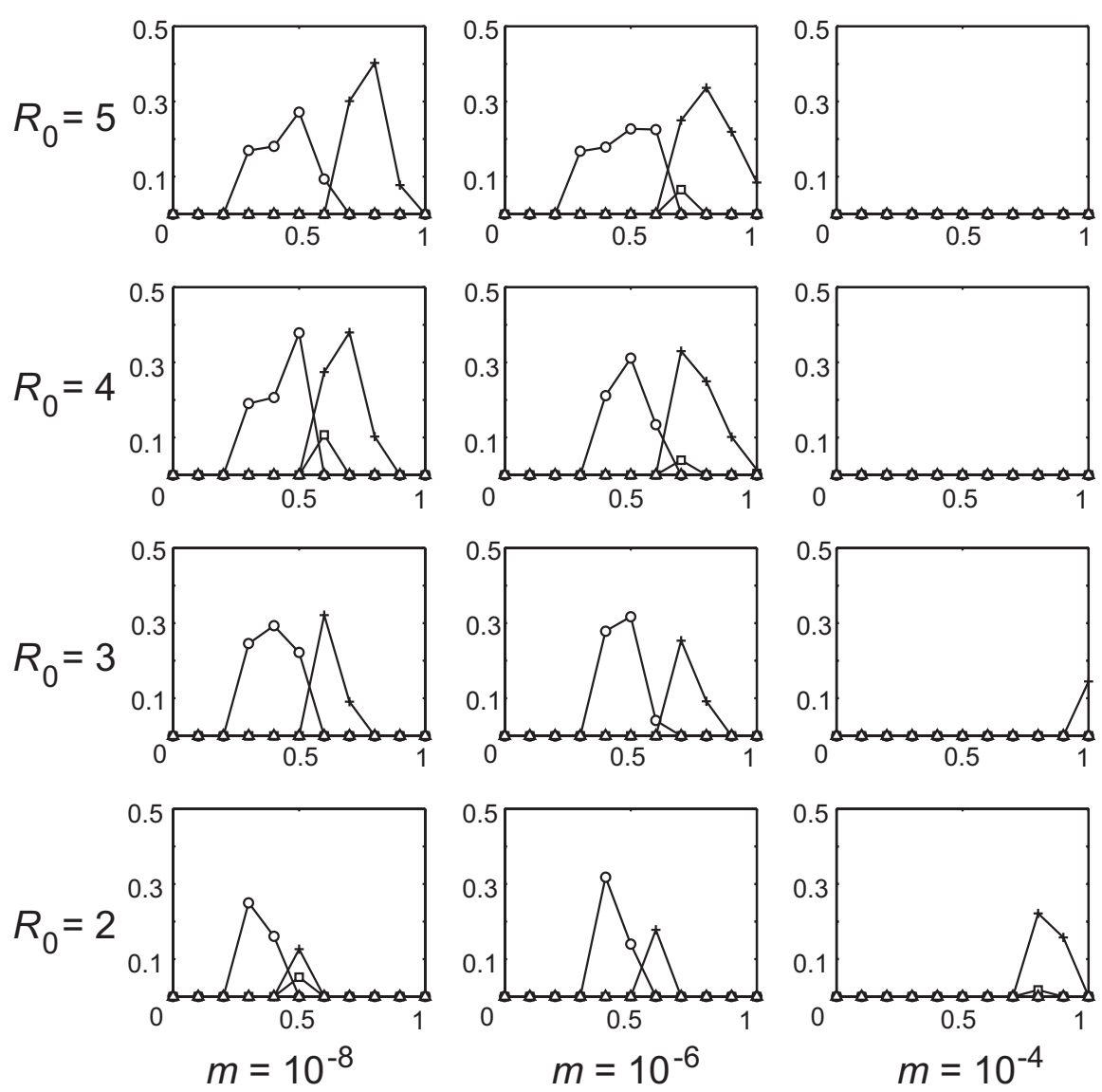

Figure S2: Qualitative accuracy measure ρ for the performance of four models with coinfections on the Topology 2 system: Gog and Grenfell's model (circles), order-1 independence closure (pluses), order-1 interpolation closure (squares), order-2 independence closure (triangles). Plots in each row have the value of R_0 that is indicated on the left; plots in each column have the value of m that is indicated at the bottom.

$$\sigma = \begin{pmatrix} 1 & s & s & s & 0 & 0 \\ s & 1 & s & 0 & 0 & 0 \\ s & s & 1 & 0 & 0 & s \\ s & 0 & 0 & 1 & s & s \\ 0 & 0 & 0 & s & 1 & s \\ 0 & 0 & s & s & s & 1 \end{pmatrix}.$$

Model with coinfections

According to the procedure outlined in the main text, we numerically find solutions for topologies 2, 3 and 4. The results are shown in Figures S2 – S7.

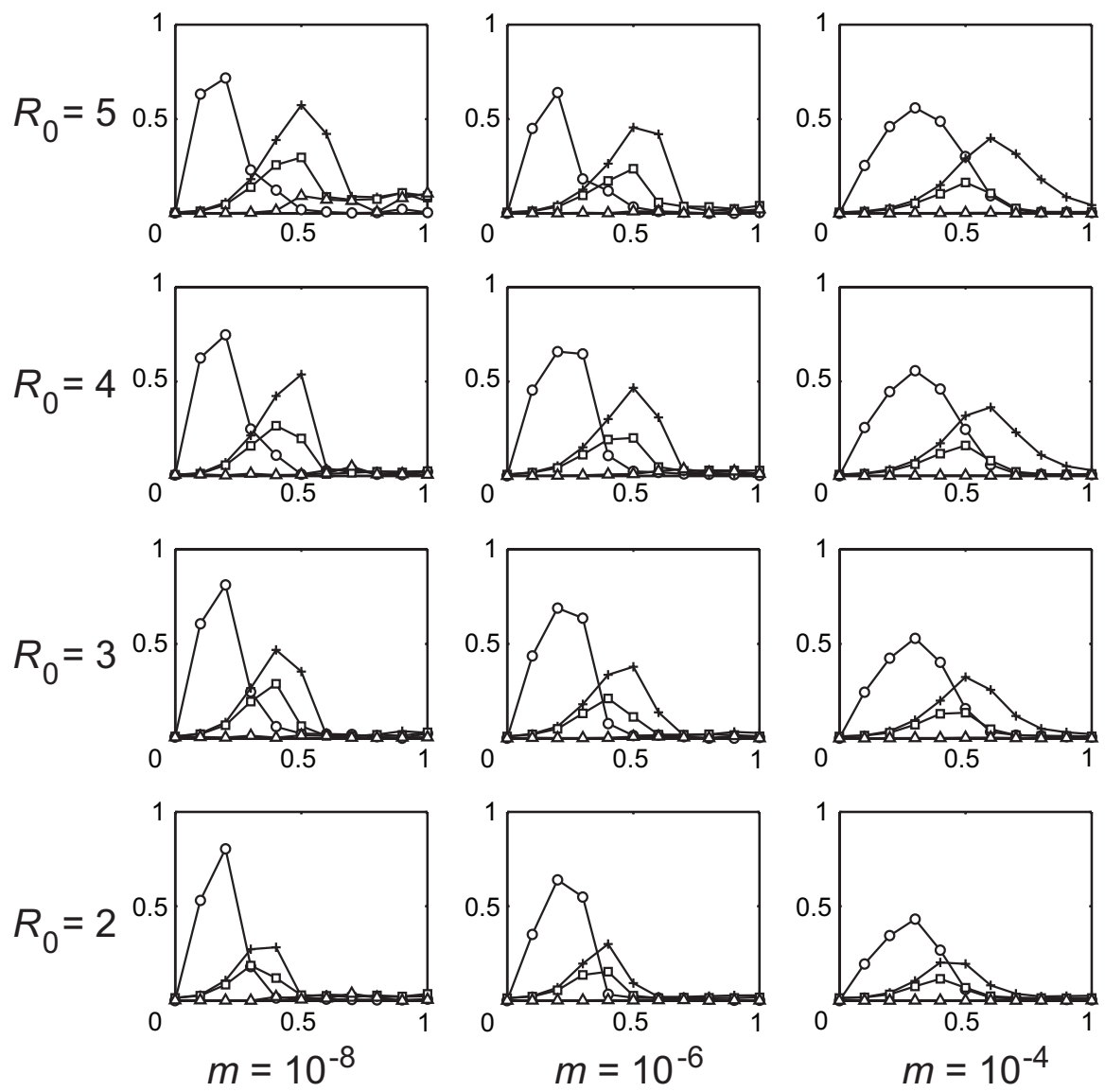

Figure S3: Quantitative accuracy measure Δ for the performance of four models with coinfections on the Topology 2 system. Details as in Figure S2.

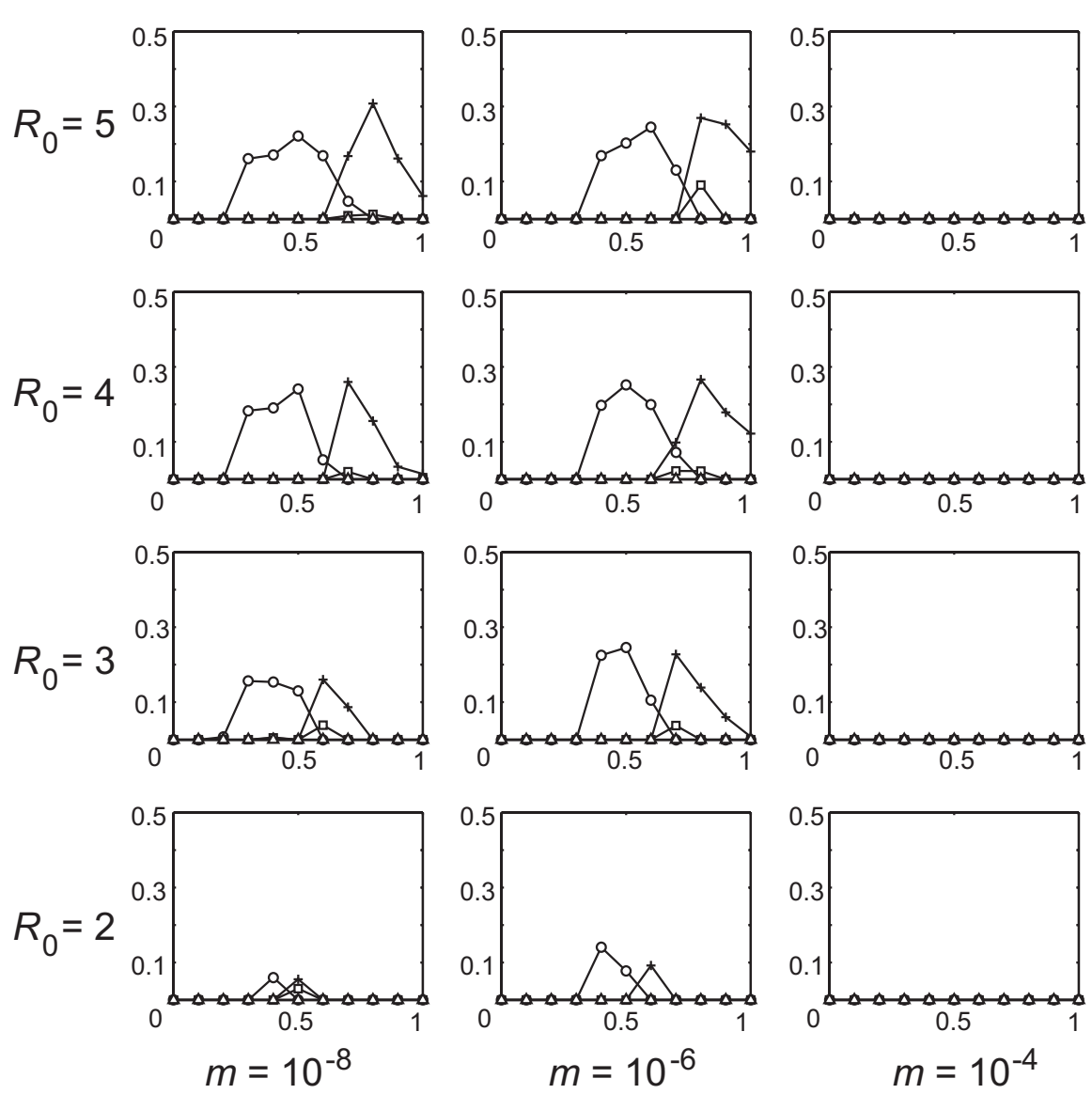

Figure S4: Qualitative accuracy measure ρ for the performance of four models with coinfections on the Topology 3 system. Details as in Figure S2.

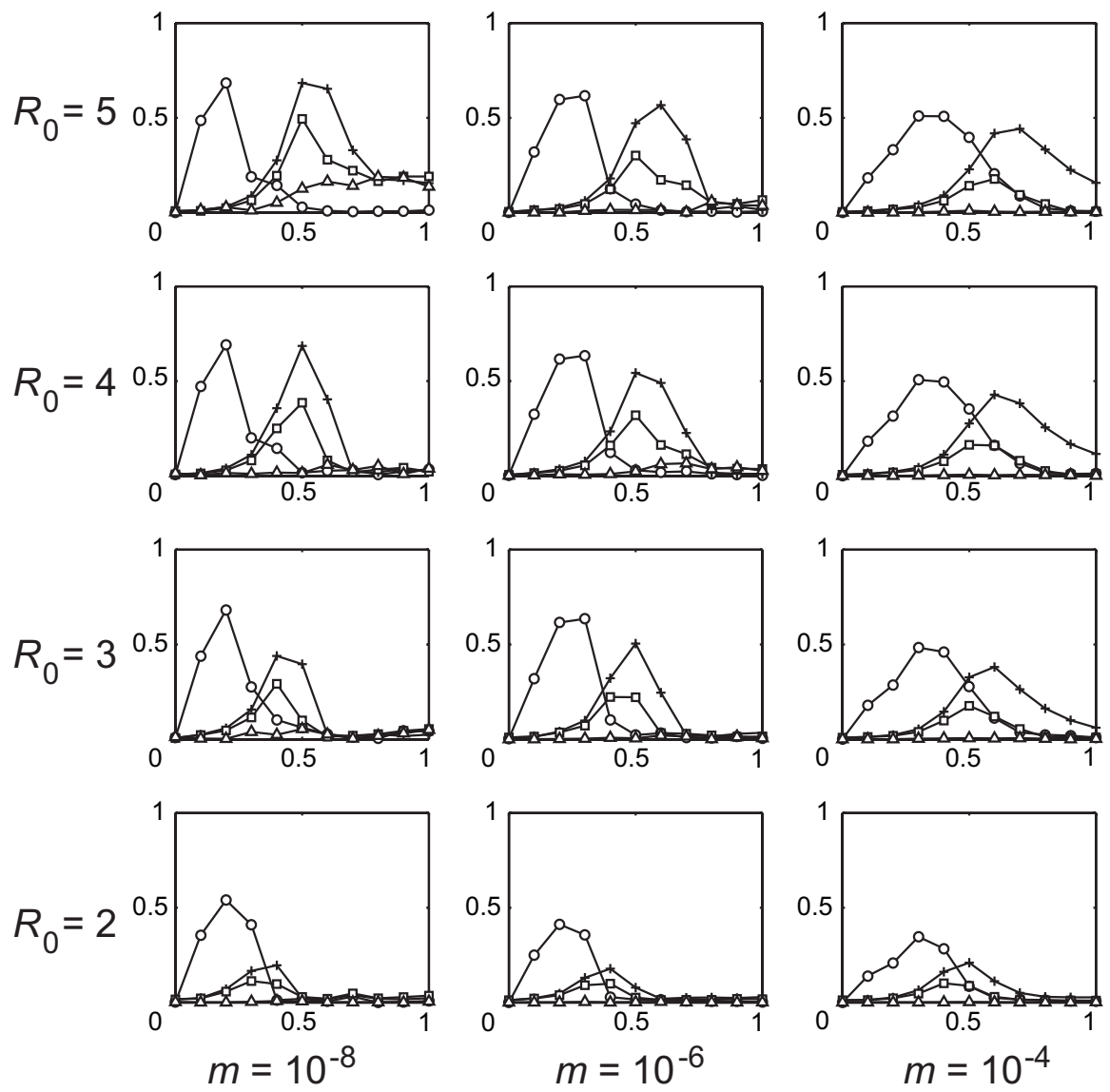

Figure S5: Quantitative accuracy measure Δ for the performance of four models with coinfections on the Topology 3 system. Details as in Figure S2.

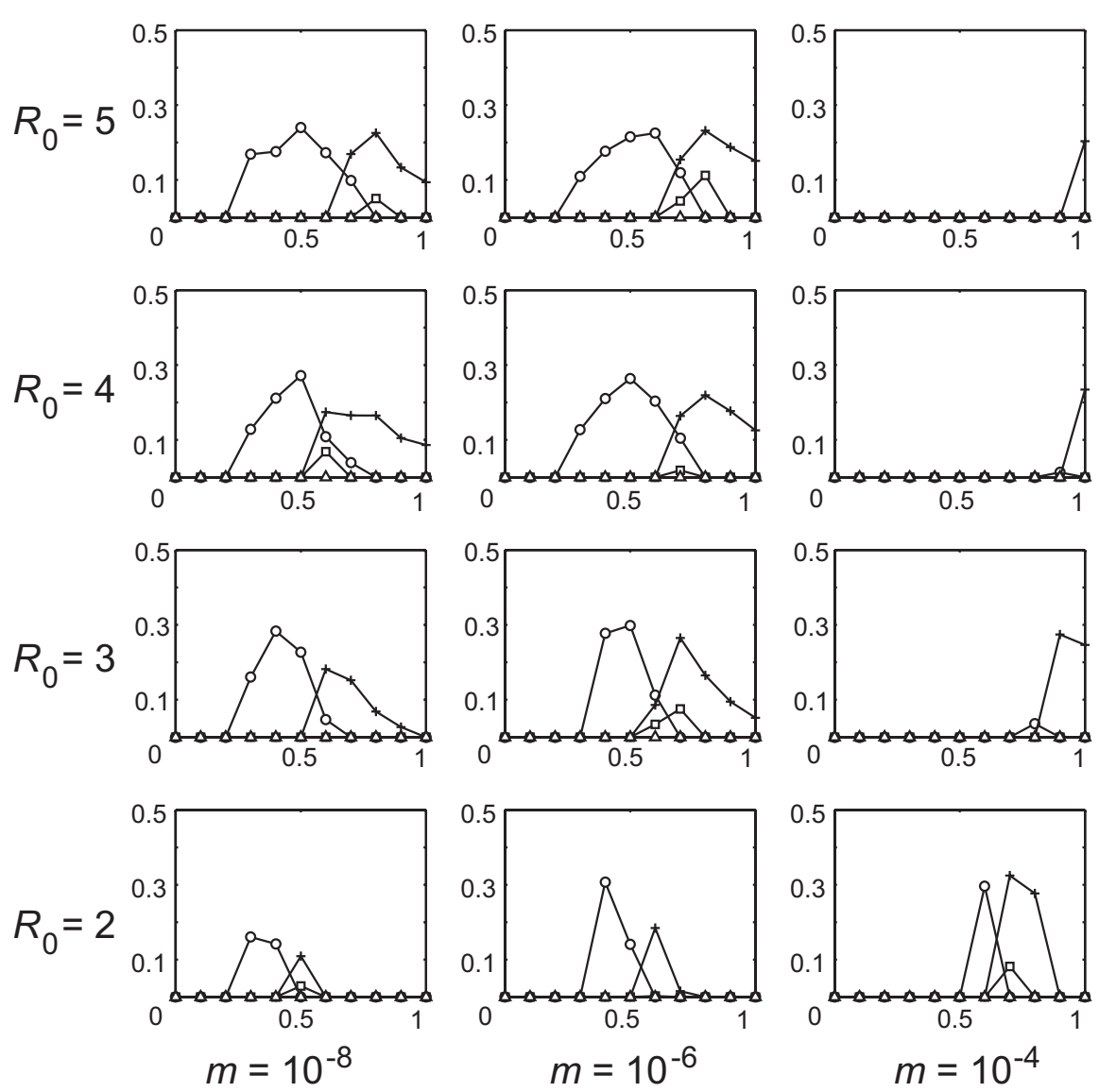

Figure S6: Qualitative accuracy measure ρ for the performance of four models with coinfections on the Topology 4 system. Details as in Figure S2.

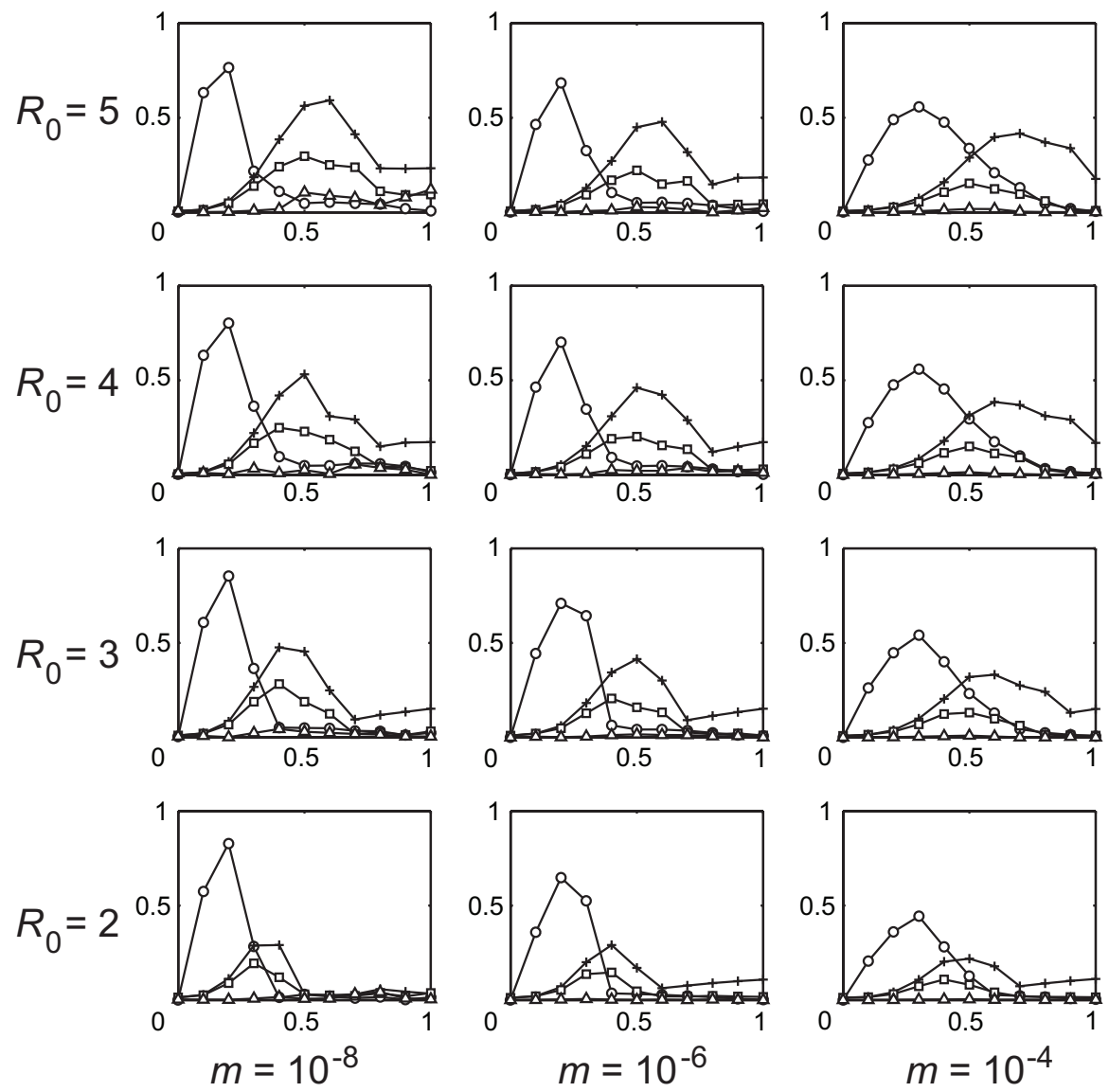

Figure S7: Quantitative accuracy measure Δ for the performance of four models with coinfections on the Topology 4 system. Details as in Figure S2.

Model with no coinfections

For the model with no coinfections, we numerically solve the following equations for the time interval $[0, T]$ and for a range of parameters:

1. The full SIR system: equations (S8) and (S9).
2. The approximation based on the order-1 independence closure: equations (S11), (S12), and (S16), where the ξ_{ij} are substituted according to (S18).
3. The approximation based on the order-1 interpolation closure: equations (S11), (S12), and (S16), where the ξ_{ij} are substituted according to (S19).
4. The approximation based on the order-2 independence closure: equations (S11), (S12), (S13), (S16), and (S17), where the ξ_{ijk} are substituted according to (S20).

Initially, 99% of the host population are healthy and fully susceptible to all strains, while 1% is infected with strain 1. All other parameter values are the same as in the model with coinfections. The results for topologies 1 – 4 are shown in Figures S8 – S15.

Additional analysis of the influenza A drift model

In this section we present results of additional simulations for our influenza A drift model.

First, we show that, a model with homogeneous transmission coefficients exhibits a succession of diagonal variants but no coexistence (Figure S16). Therefore, heterogeneity in transmission coefficients is necessary in our model to ensure coexistence of similar

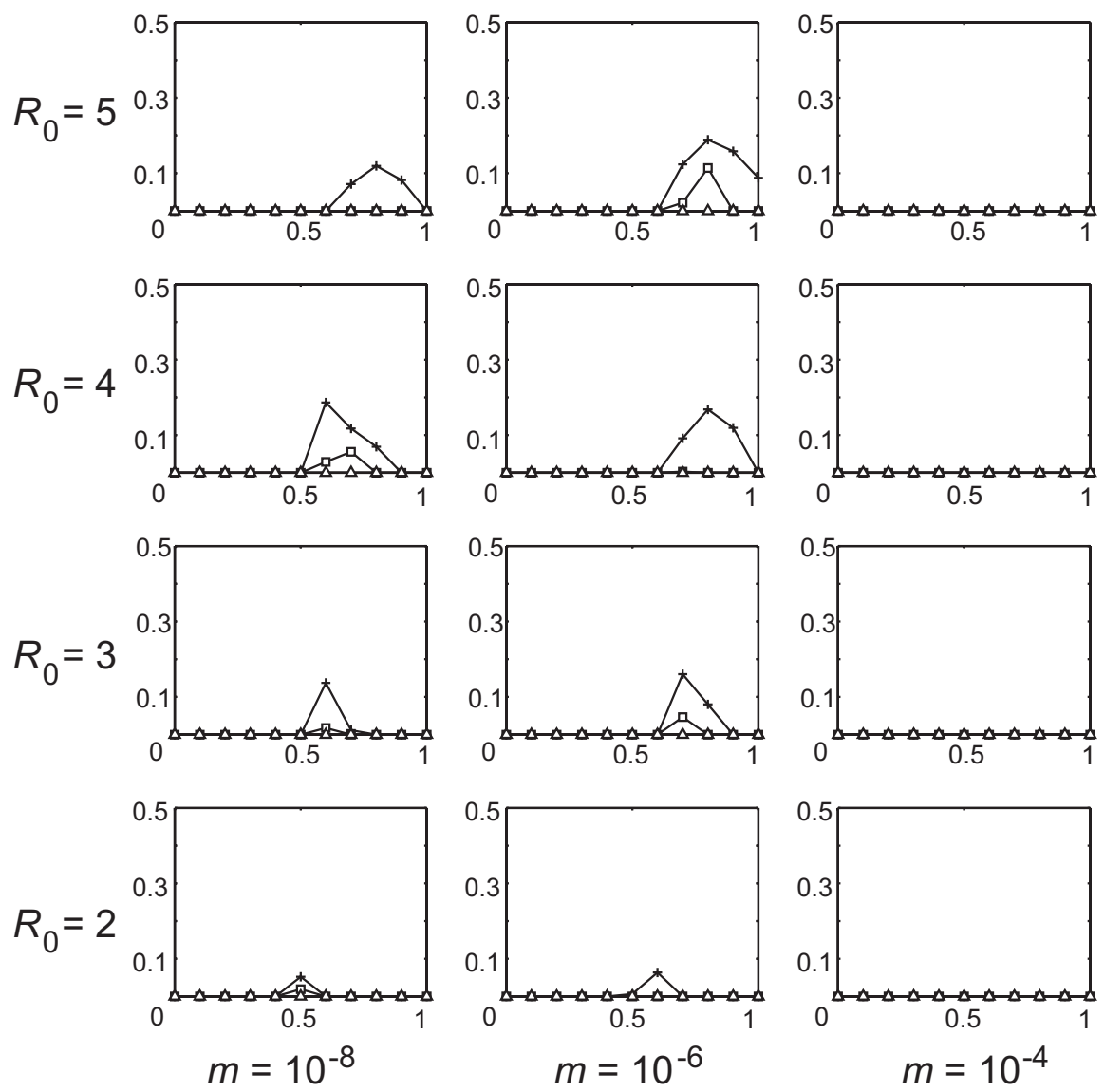

Figure S8: Qualitative accuracy measure ρ for the the performance of three models with no coinfections on the Topology 1 system: order-1 independence closure (pluses), order-1 interpolation closure (squares), order-2 independence closure (triangles). Plots in each row have the value of R_0 that is indicated on the left; plots in each column have the value of m that is indicated at the bottom.

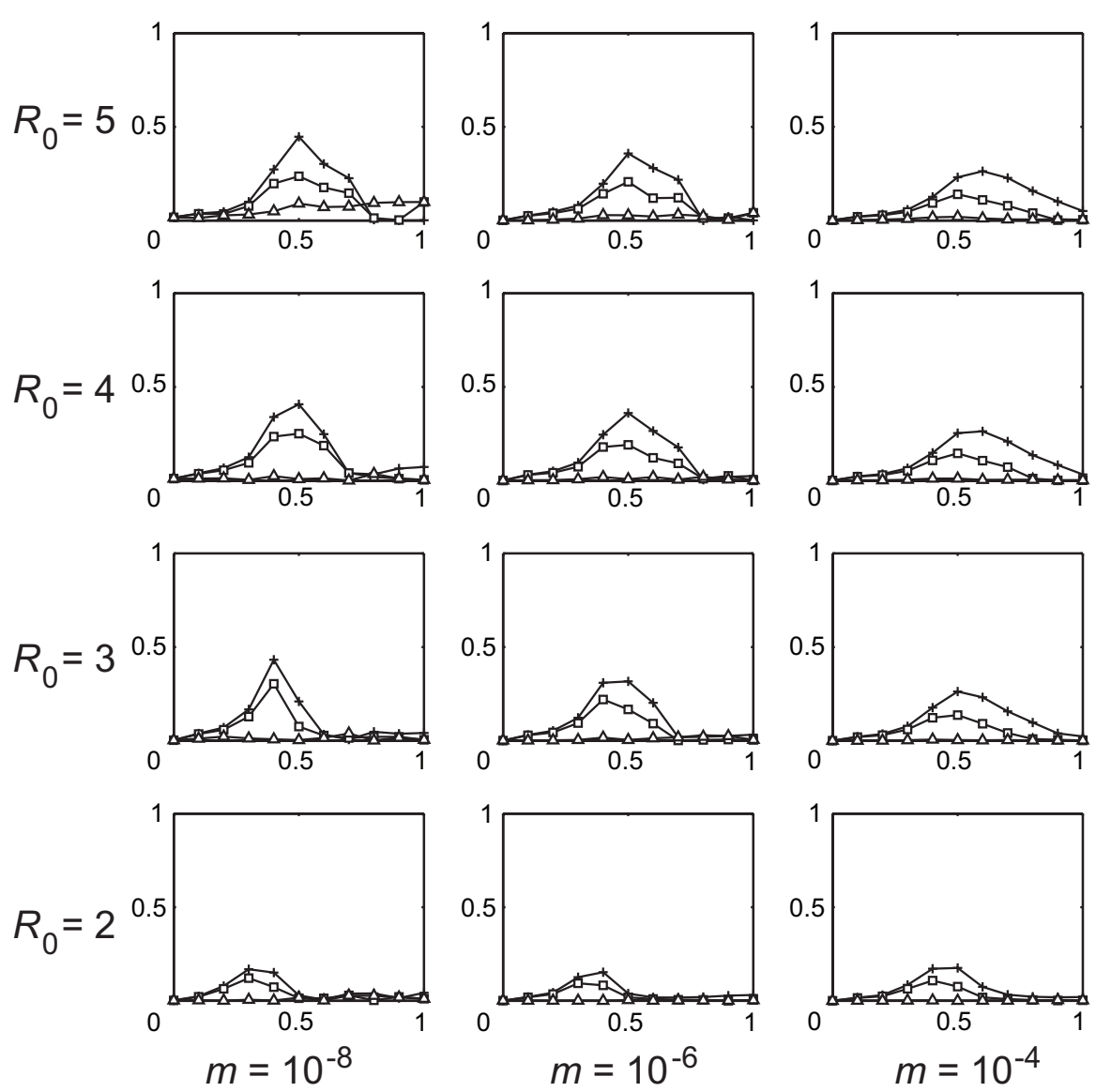

Figure S9: Quantitative accuracy measure Δ for the performance of three models with no coinfections on the Topology 1 system. Details as in Figure S8.

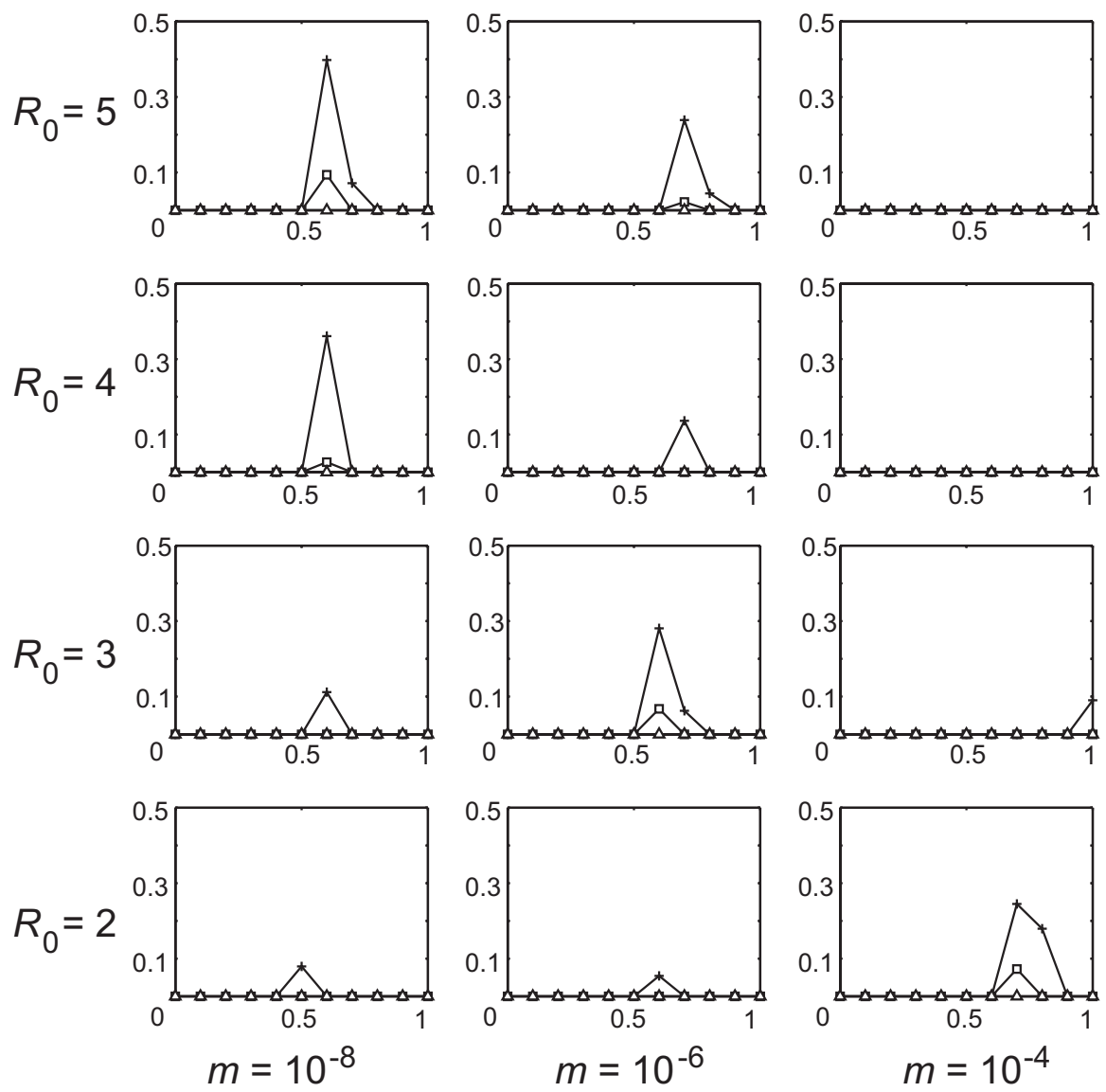

Figure S10: Qualitative accuracy measure ρ for the the performance of three models with no coinfections on the Topology 2 system. Details as in Figure S8.

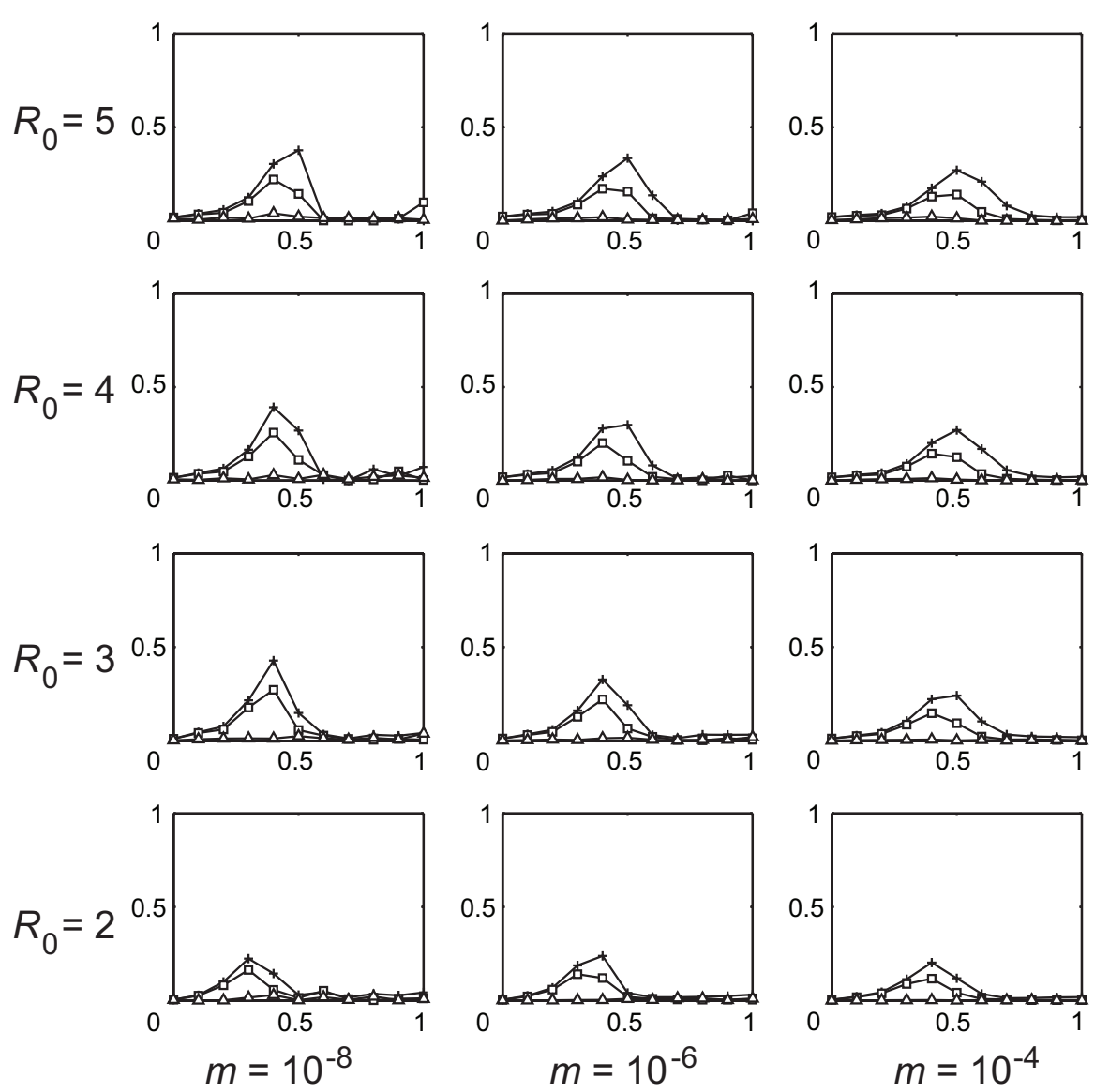

Figure S11: Quantitative accuracy measure Δ for the performance of three models with no coinfections on the Topology 2 system. Details as in Figure S8.

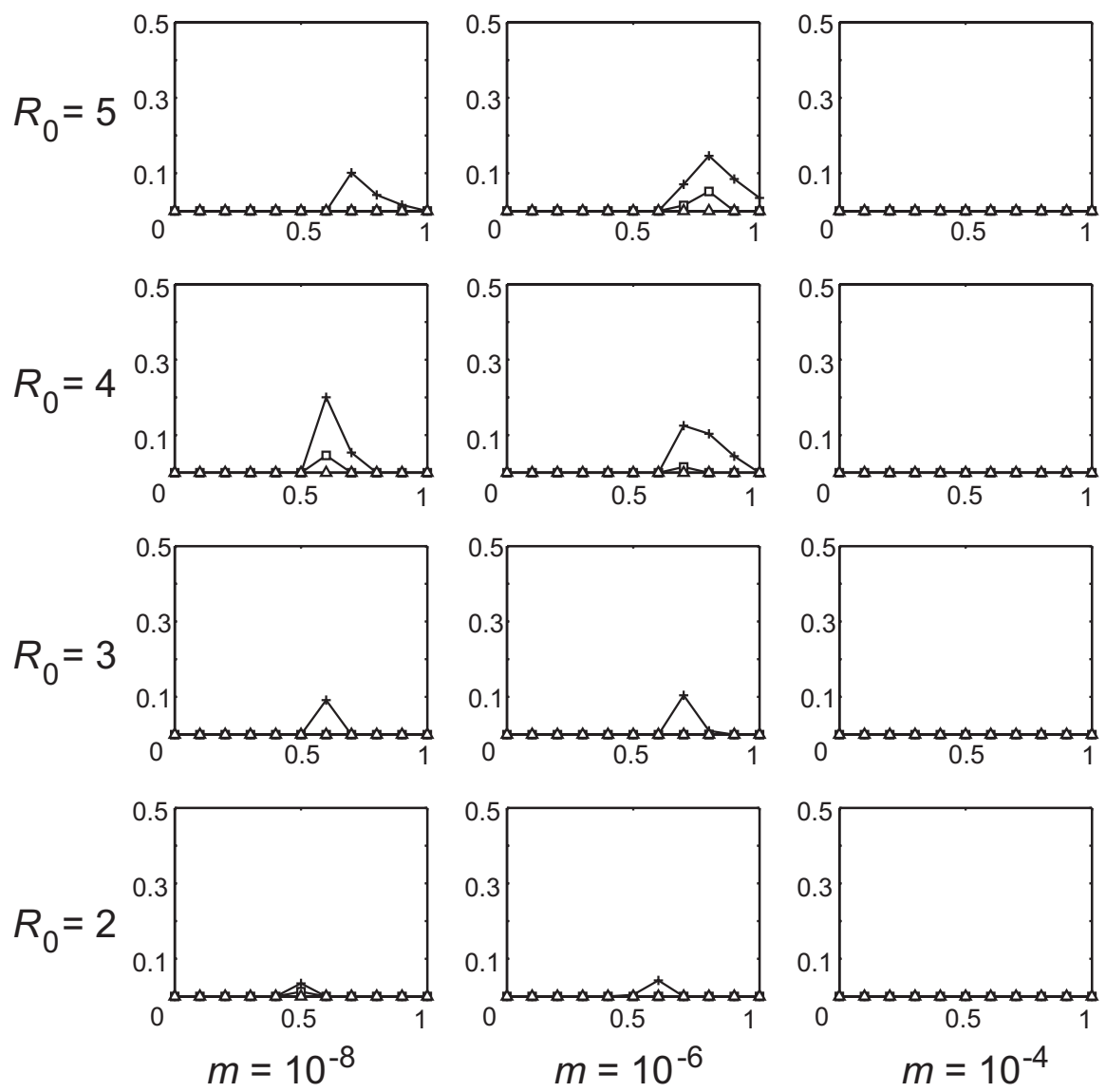

Figure S12: Qualitative accuracy measure ρ for the the performance of three models with no coinfections on the Topology 3 system. Details as in Figure S8.

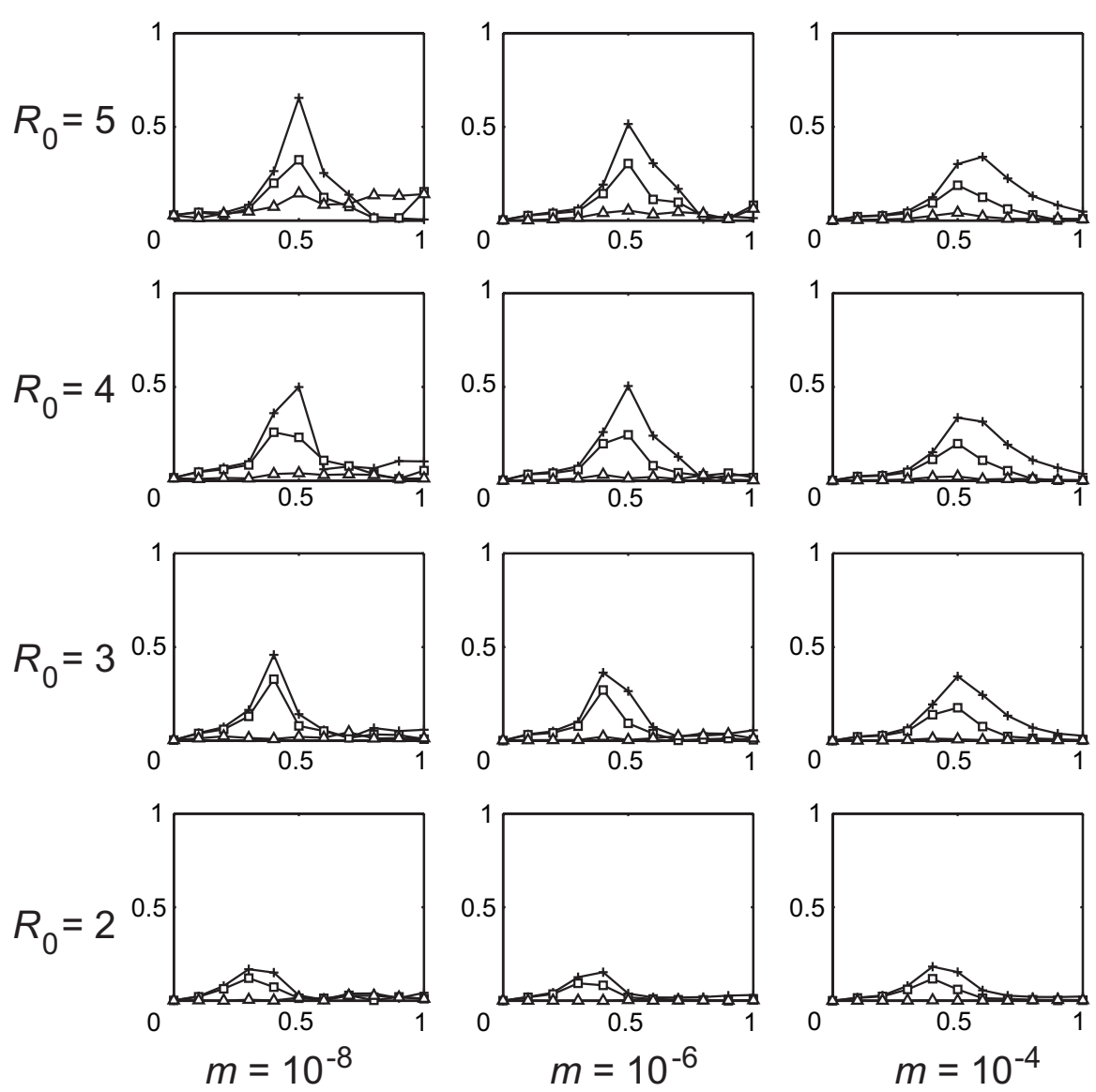

Figure S13: Quantitative accuracy measure Δ for the performance of three models with no coinfections on the Topology 3 system. Details as in Figure S8.

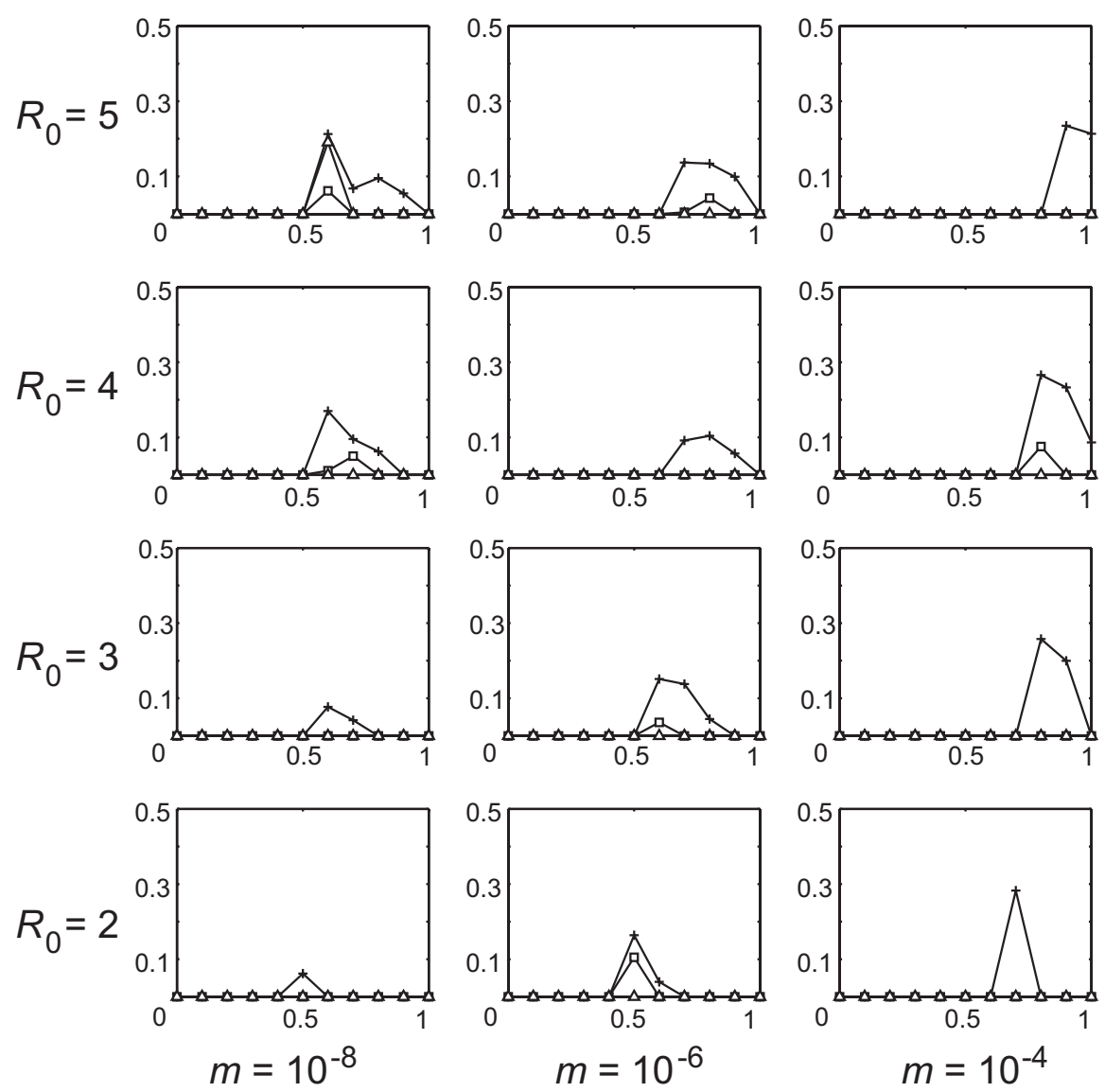

Figure S14: Qualitative accuracy measure ρ for the the performance of three models with no coinfections on the Topology 4 system. Details as in Figure S8.

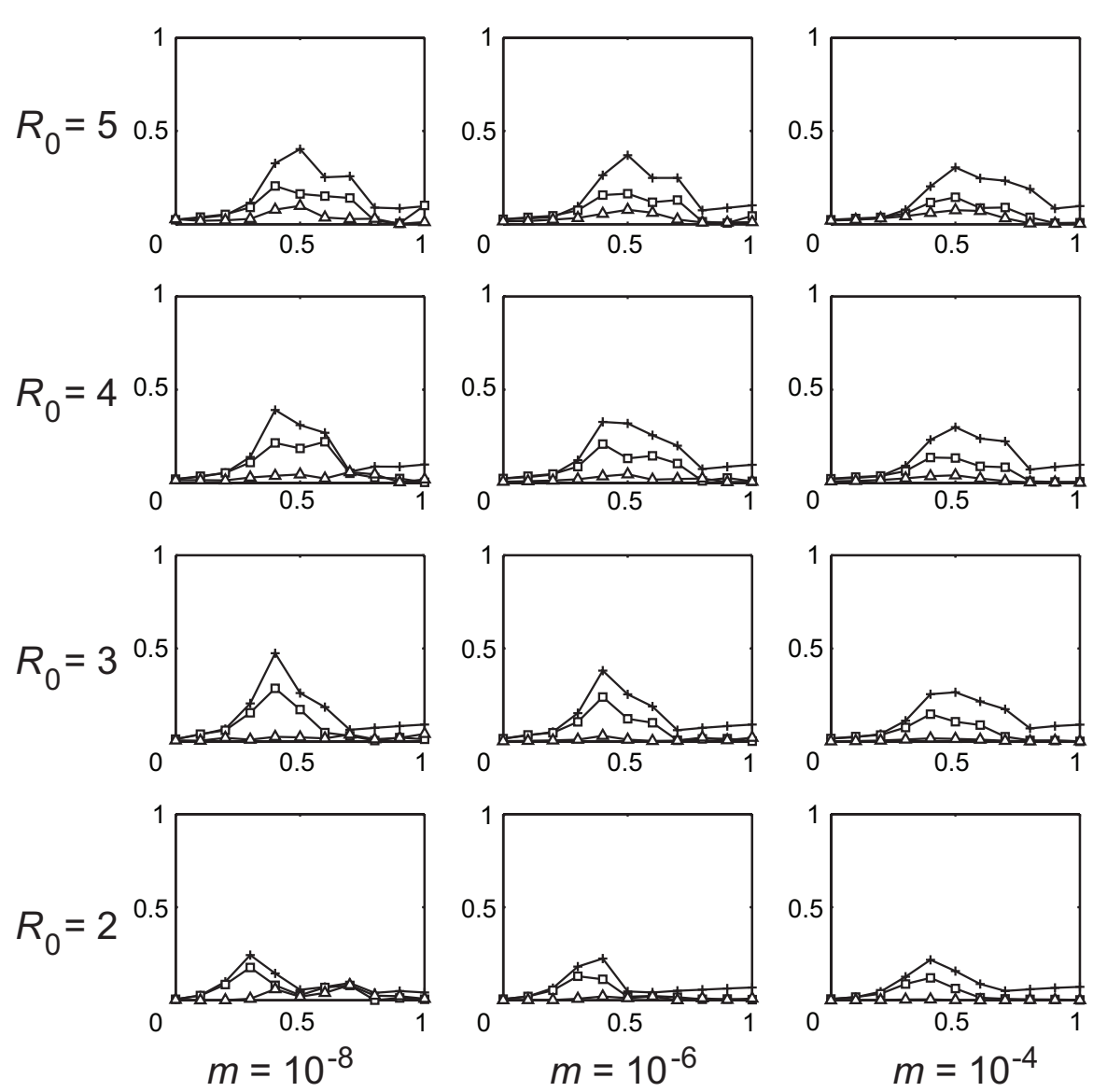

Figure S15: Quantitative accuracy measure Δ for the performance of three models with no coinfections on the Topology 4 system. Details as in Figure S8.

variants. It is interesting to notice that in the model by Tria et al. [S3] heterogeneity in transmission coefficients had an opposite effect – it was necessary to constrain viral diversity. Although that model is qualitatively different from ours in many respects and cannot be compared to our model directly, we think that the main reason for this discrepancy is the fact that Tria et al. assume local cross-immunity structure (in their model the strength of cross-immunity monotonically depends on the Hamming distance between variant sequences) while we assume highly non-local cross-immunity structure. We conjecture that the effect of heterogeneity of transmission coefficients in Gog and Grenfell's model [S1] with strain-independent cross-immunity would coincide with that by Tria et al.

The second simulation differs from the model described in main text only by the shape of the cross-immunity structure between strains. We demonstrate that, if cross-immunity structure is local, as shown in Figure 4B in the main text, constrained evolution is impossible – the virus explores the whole strain space, unless, of course, some part of it is unviable, i.e., transmission coefficients are equal to or less than unity, as in Gog in Grenfell's work [S1]. Here we use essentially the same local cross-immunity structure as Gog and Grenfell [S1]:

$$\sigma^{(\mathrm{loc})}_{(i,j)(k,\ell)} = \exp\left\{ -\frac{1}{2}\left(\frac{|i-k|+|j-\ell|}{a}\right)^2 \right\}.$$

The results of this simulation are shown in Figure S17. Note that the sum of fractions of infected hosts surpasses 1 and reaches its maximum near 5 before it starts dropping. This implies that many hosts survive multiple coinfections. Obviously, this does not happen in a model where coinfections are prohibited, despite an explosion in diversity that is still captured (results not shown). Also note that the decline in the number of coinfections in the second half of the simulation is probably due to the fact that the whole 20 by 20 strain space was explored within the simulation time.

The final set of simulations aims at demonstrating that the main qualitative result of our paper – the one-dimensionality of influenza A drift – does not depend on the details of the model. We simulate the influenza evolution in a setting with the immunity structure and the distribution of transmission coefficients as described in the main text using now Gog and Grenfell's model (Figure S18) and the approximate model with no coinfections with the order-1 interpolation closure (Figure S19). As expected, the evolution of the virus is principally one-dimensional irrespectively of the model. However, it is instructive to notice some similarities and differences between the simulation results in different models:

1. The sets of strains that cause epidemics coincide for both our models but slightly differ from the set predicted by Gog and Grenfell's model (for example, in the latter model, strains (12,7) or (16,16) do not cause epidemics). In general, strains cause less severe epidemics in Gog and Grenfell's model than in either of our models. This is consistent with the fact that Gog and Grenfell's model overestimates the level of immunity in the population.

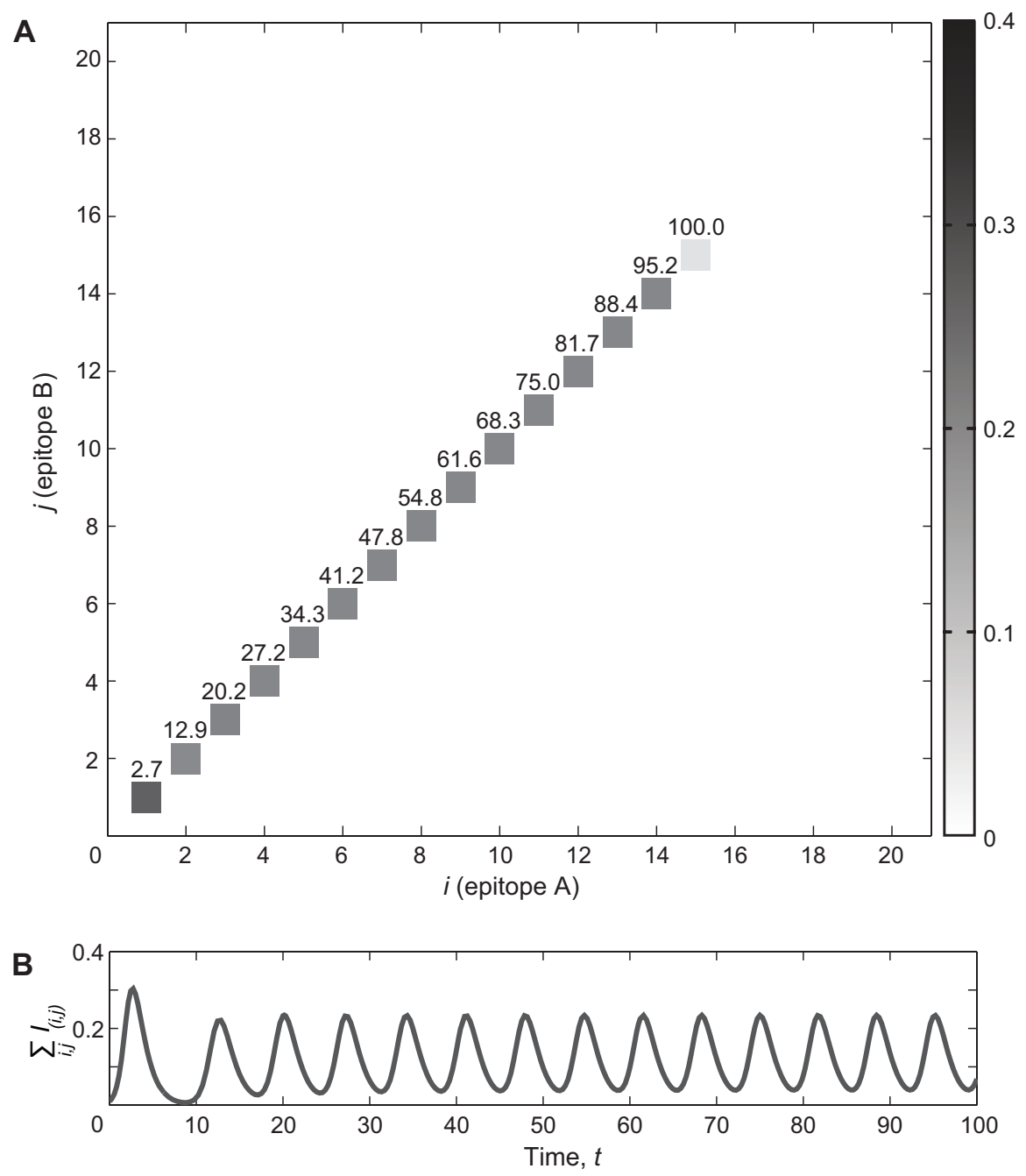

Figure S16: Approximate dynamics of antigenic drift in influenza A, based on the order-1 interpolation closure in a model with coinfections. All transmission coefficients $\beta_{(i,j)}$ are equal to 3. Other parameter values are the same as in Figure 5 in the main text.

2. The epidemic peak times in our model with coinfections generally coincide with those in Gog and Grenfell's model, while the corresponding epidemics occur later in the model where coinfections are excluded. This suggests that the evolution of the virus proceeds slower in a system with no coinfections.

These observations provide additional evidence for the fact that, even though the coarse qualitative behavior of status-based models based on different assumptions may be quite similar even in complex settings, substantial quantitative differences in predictions of such models do exist.

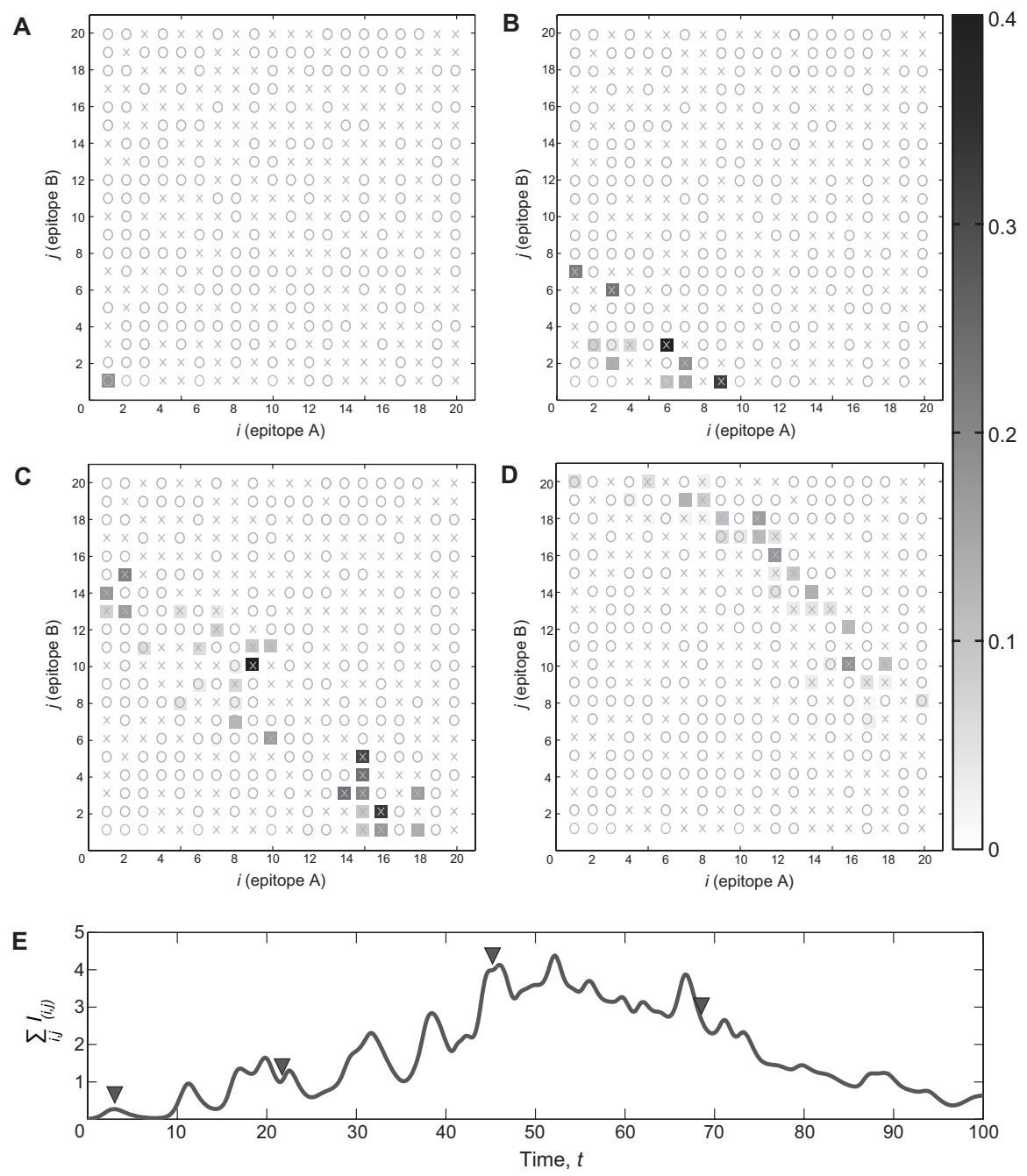

Figure S17: Approximate dynamics of antigenic drift in influenza A, based on the order-1 interpolation closure in a model with coinfections. $\sigma = \sigma^{(\mathrm{loc})}$. All other parameters, including parameter a and the landscape of transmission coefficients are the same as in Figure 5 in the main text. Four snapshots are taken at times $t = 3$ (**A**), $t = 23$ (**B**), $t = 46$ (**C**), and $t = 67$ (**D**). **E**. The sum of all proportions of infectious hosts as a function of time.

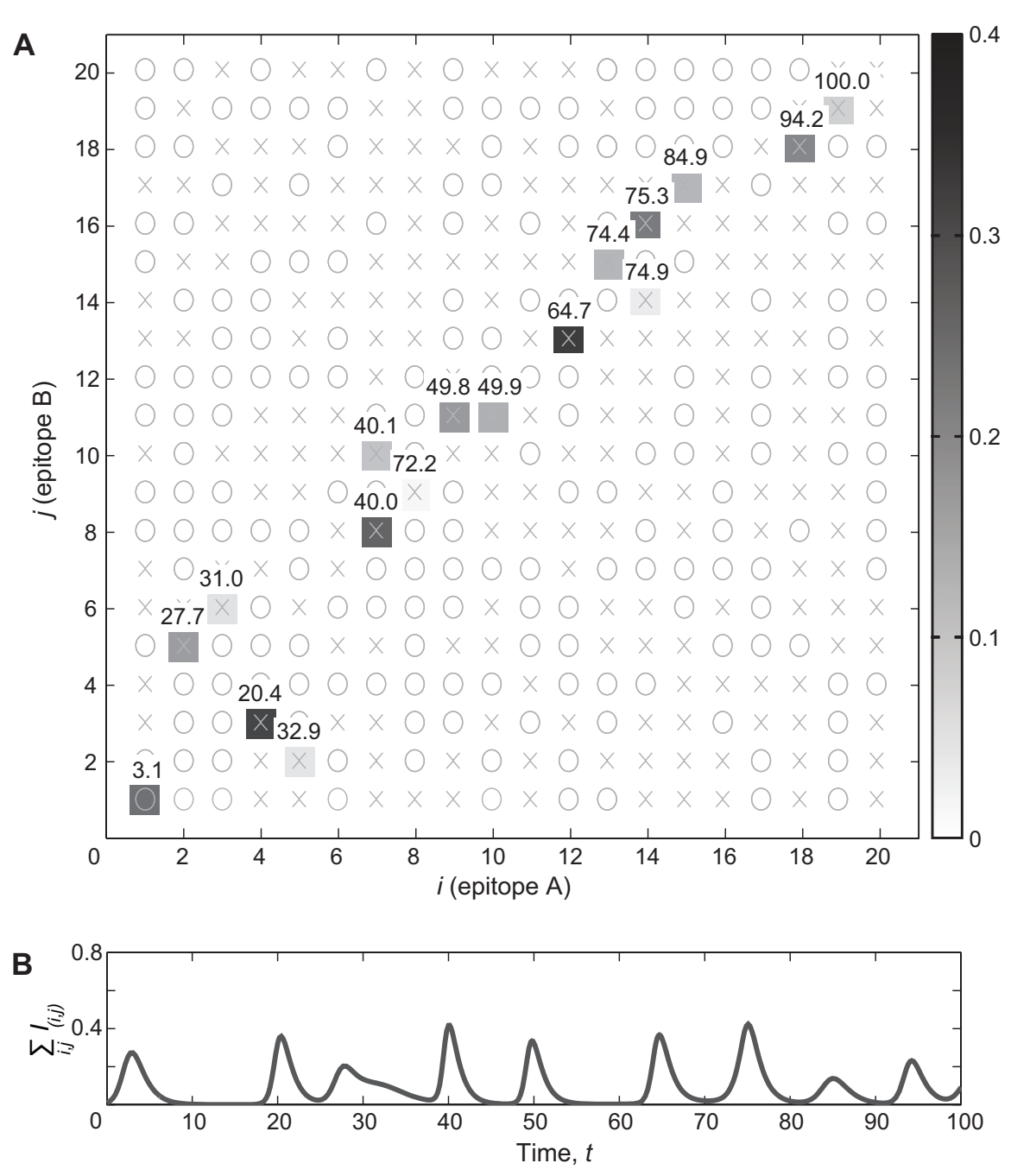

Figure S18: Approximate dynamics of antigenic drift in influenza A, based on Gog and Grenfell's model. All parameters, including the landscape of transmission coefficients are the same as in Figure 5 in the main text.

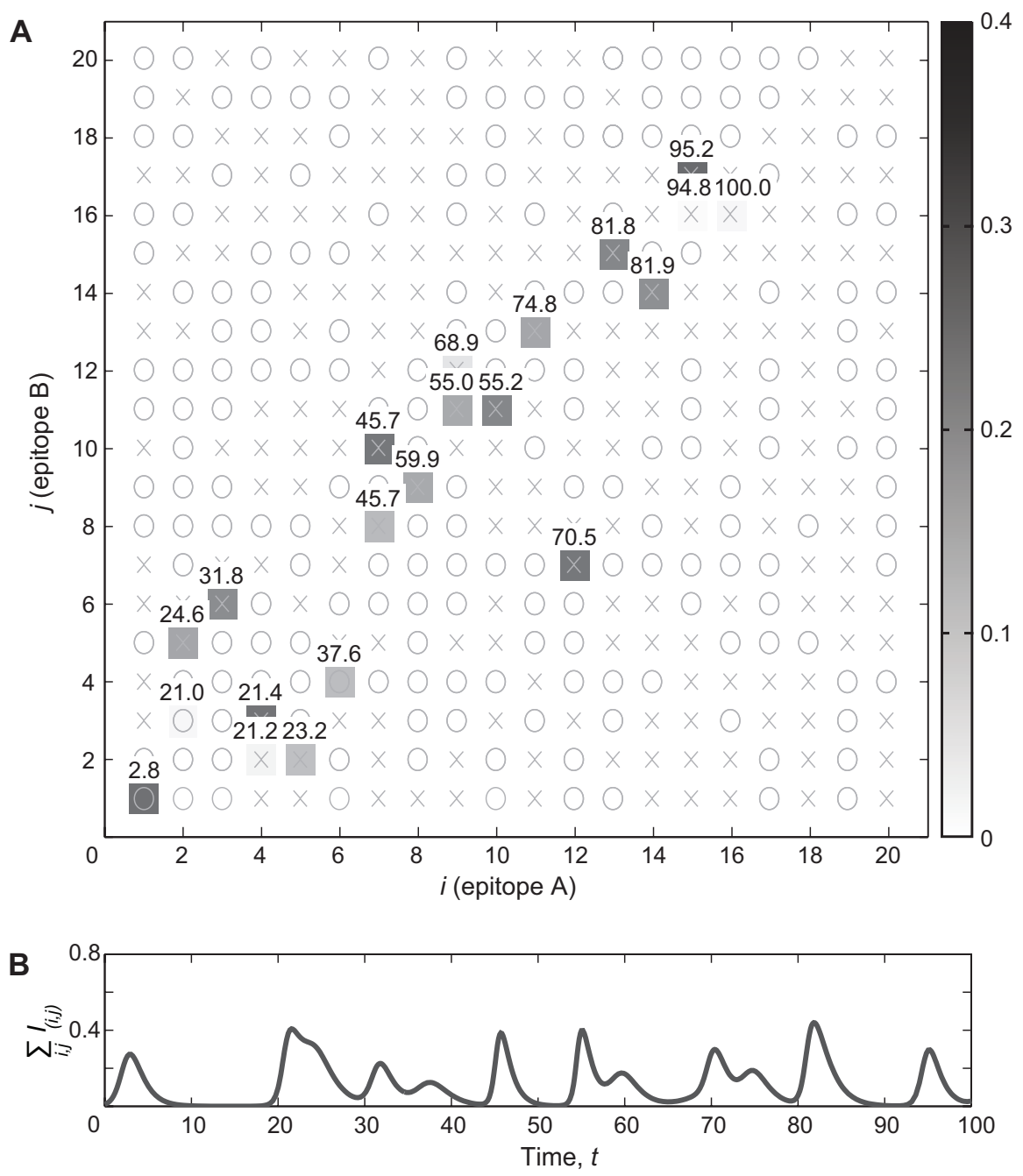

Figure S19: Approximate dynamics of antigenic drift in influenza A, based on the order-1 interpolation closure in a model with no coinfections. The initial condition was given by all state variables being zero except for $I_{(1,1)}(0) = 0.01$, corresponding to a healthy and fully susceptible host population with 1% of hosts infected with strain $(1, 1)$. All other parameters including the landscape of transmission coefficients are the same as in Figure 5 in the main text. **B**. The total fraction of infected hosts as a function of time.

References

[S1] Gog JR, Grenfell BT (2002) Dynamics and selection of many-strain pathogens. Proc Natl Acad Sci USA 99:17209–17214.

[S2] Gog JR, Swinton J (2002) A status-based approach to multiple strain dynamics. J Math Biol 44:169–184.

[S3] Tria F, Lässing M, Peliti L, Franz S (2005) A minimal stochastic model for influenza evolution. J Stat Mech P07008.
